# Swip-1 promotes exocytosis of glue granules in the exocrine *Drosophila* salivary gland

**DOI:** 10.1242/jcs.260366

**Published:** 2023-03-06

**Authors:** Franziska Lehne, Sven Bogdan

**Affiliations:** Institute of Physiology and Pathophysiology, Department of Molecular Cell Physiology, Philipps-University Marburg, 35037 Marburg, Germany

**Keywords:** Swip-1, *Drosophila*, Salivary gland, Exocytosis, Actin, Cross-linker, Ca^2+^, EF-hand domain, Secretion

## Abstract

Exocytosis is a fundamental cellular process by which cells secrete cargos from their apical membrane into the extracellular lumen. Cargo release proceeds in sequential steps that depend on coordinated assembly and organization of an actin cytoskeletal network. Here, we identified the conserved actin-crosslinking protein Swip-1 as a novel regulator controlling exocytosis of glue granules in the *Drosophila* salivary gland. Real-time imaging revealed that Swip-1 is simultaneously recruited with F-actin onto secreting granules in proximity to the apical membrane. We observed that Swip-1 is rapidly cleared at the point of secretory vesicle fusion and colocalizes with actomyosin network around the fused vesicles. Loss of Swip-1 function impairs secretory cargo expulsion, resulting in strongly delayed secretion. Thus, our results uncover a novel role of Swip-1 in secretory vesicle compression and expulsion of cargo during regulated exocytosis. Remarkably, this function neither requires Ca^2+^ binding nor dimerization of Swip-1. Our data rather suggest that Swip-1 regulates actomyosin activity upstream of Rho-GTPase signaling to drive proper vesicle membrane crumpling and expulsion of cargo.

## INTRODUCTION

Regulated exocytosis is an important mechanism by which secretory cells package molecules in secretory vesicles and deliver them to the extracellular environment. It is a stepwise process that involves the biogenesis of secretory vesicles, their transport to the cell periphery, their fusion with the apical membrane and the release of vesicle content to the outside (Burgoyne and Morgan, 2003; Spiliotis and Nelson, 2003; Wu et al., 2014). These processes highly depend on a cortical actin cytoskeleton that forms a dense network associated with the plasma membrane (Nightingale et al., 2012; Porat-Shliom et al., 2013). Previous studies showed distinct functions of the actin cortex in regulated exocytosis (Nightingale et al., 2012; Trifaro et al., 2008). Secretory vesicles form an actin coat that acts as a physical barrier preventing premature vesicle fusion, but, together with myosin II (also known as Zip), provides the mechanical forces for vesicle compression and expulsion of cargo into the extracellular lumen (Masedunskas et al., 2011; Nightingale et al., 2011; Sokac et al., 2003).

The *Drosophila* salivary gland is a typical exocrine gland consisting of two interconnected monolayered tubes with columnar epithelial cells on each side that secrete high levels of glue proteins, important for the attachment of the pupa to a surface, which also release glycosylated mucin or non-digestive enzymes for lubricating food (Abrams et al., 2003; Biyasheva et al., 2001). More recent studies further established the *Drosophila* salivary gland as a powerful *ex vivo* 3D imaging model system to dissect the molecular mechanism of the actin coat formation and actomyosin-dependent vesicle compression of single exocytic events at high resolution (Rousso et al., 2016; Tran et al., 2015). Both studies provided strong evidence for essential roles of branched actin nucleators such as the Arp2/3 complex and its activator WASP in the process of secretory cargo expulsion and integration of vesicular membranes with the apical plasma membrane (Rousso et al., 2016; Tran et al., 2015). Secretion is initiated by the clearance of apical F-actin at the plasma membrane, followed by fusion pore formation and subsequent directional F-actin recruitment to the fused secretory vesicle membrane (Merrifield, 2016; Tran and Ten Hagen, 2017). Actin assembly on the vesicular membrane is driven by sequential recruitment of actin nucleators. Previous studies suggested that it starts with the recruitment of Diaphanous (Dia) and its activator Rho promoting nucleation of linear actin filaments on vesicle surface, followed by Arp2/3- and WASP-branched actin polymerization, first preventing premature vesicle fusion, and eventually promoting myosin II-dependent vesicle compression and expulsion (Merrifield, 2016; Tran and Ten Hagen, 2017).

Here, we identified the Swip-1 protein as a novel regulatory component of exocytosis in *Drosophila* salivary glands. Members of the Swip-1 protein family are conserved actin-binding proteins with two EF-hand domains and one coiled-coil domain at the C-terminus. Previous studies have implicated Swip-1 in diverse cellular processes including adhesion turnover, cell spreading and migration, endocytosis, B-cell receptor signaling and cancer invasion (Kwon et al., 2013; Moreno-Layseca et al., 2021; Reimer et al., 2020; Tu et al., 2018; Zhang et al., 2018). A recent study identified Swip-1 as an important Ca^2+^-dependent actin-crosslinking protein driving rapid reorganization of actin networks in lamellipodial protrusions and epithelial wound closure (Lehne et al., 2022). In this work, we found that Swip-1 is recruited to secreting granules in proximity to the apical membrane of salivary glands. Swip-1 is rapidly cleared at the point of secretory vesicle fusion and colocalizes with the actin cytoskeleton network around the fused vesicles. Loss of Swip-1 function impairs secretory cargo expulsion, resulting in strongly delayed secretion. Our data further suggest that Swip-1 is not required for vesicle fusion, but rather implicated in actomyosin-mediated contractility on the vesicle membrane driving membrane crumpling and cargo release.

## RESULTS

### Swip-1 is highly expressed in salivary gland tissue and accumulates at fused secretory vesicles upon ecdysone induction

**Fig. 1.**
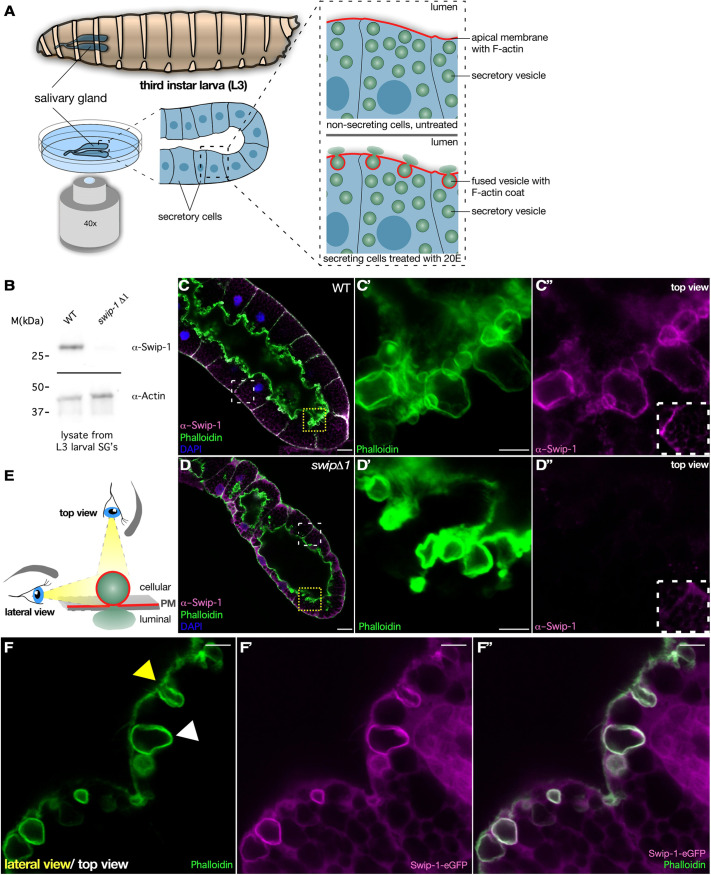
**Localization of Swip-1 at the apical membrane and secreting vesicles in salivary glands.** (A) Left: schematic overview of imaging setup to visualize exocytosis in *ex vivo* larval salivary glands. Right: detailed schemes of non-secreting and 20-hydroxyecdysone (20E)-induced secreting epithelial cells showing F-actin localization at the apical membrane and around fusing vesicles. (B) Western blot analysis of salivary gland lysates of wild-type (WT) and *swip-1* mutant larvae. Knockout was validated with an anti-Swip-1-specific antibody. Actin served as loading control. (C) Confocal image of a fixed wild-type salivary gland stained for DAPI (blue), F-actin (Phalloidin, green) and Swip-1 (magenta). Swip-1 is located in the cytoplasm and enriched at the plasma membrane. Scale bar: 25 µm. (C′,C″) Detailed views of boxed area in C. The boxed area in C″ shows anti-Swip-1 staining at the basal surface in wild type. Swip-1 localizes at fusing vesicles at the apical membrane and colocalizes with F-actin. Scale bar: 5 µm. (D) Confocal image of a fixed *swip-1* mutant salivary gland stained for DAPI (blue), F-actin (Phalloidin, green) and Swip-1 (magenta). Scale bar: 25 µm. (D′,D″) Detailed view of boxed area in D. The boxed area in D″ shows anti-Swip-1 staining at the basal surface in *swip-1* mutant. Swip-1 staining is absent at the F-actin-coated vesicles. Unspecific background staining at the basal surface remains. Scale bar: 5 µm. (E) Schematic of imaging angles. Secreting vesicles can either be imaged from above with the lumen underneath and not visible or from a lateral view, orthogonal to the apical membrane, allowing visualization of a cross section of the vesicle and the lumen. PM, plasma membrane. (F–F″) Confocal images of a fixed secreting salivary gland from larvae expressing Swip-1-eGFP (magenta) under the control of srp-Gal4 and stained for F-actin (green). Differences in vesicle visualization by lateral and top view are indicated by yellow and white arrowheads, respectively. Scale bars: 5 µm. Images are representative of at least three experiments.

We recently identified the EF-hand domain-containing protein Swip-1 as a conserved lamellipodial protein strongly upregulated in *Drosophila* macrophages at the onset of metamorphosis when macrophage behavior shifts from quiescent to migratory state by the steroid hormone 20-hydroxyecdysone (20E) (Lehne et al., 2022). Loss of Swip-1 function results in prominent defects in the innate immune system and epithelial wound closure, although mutant flies are fully viable without any developmental delays (Fig. S1A; Lehne et al., 2022). Gene expression data extracted from Fly Atlas 2 (Leader et al., 2018) and the Human Protein Atlas (Uhlen et al., 2015) further suggest an unknown conserved role of Swip-1 in exocrine glandular cells of the gastrointestinal tract and salivary glands. We used *ex vivo* cultured *Drosophila* third-instar larval salivary glands as an excellent model system to study the role of Swip-1 in exocytosis (Fig. 1A; Loganathan et al., 2021). Exocrine salivary glands were cultured *ex vivo* in a Petri dish and stimulated to secrete highly glycosylated mucins, so-called glue proteins, by exogenous addition of 20E (Fig. 1A; Costantino et al., 2008; Tran et al., 2015). We observed that glue proteins are stored in large secretory vesicles and released to the salivary gland lumen upon fusion with the apical plasma membrane (Fig. 1A).

**Fig. 2.**
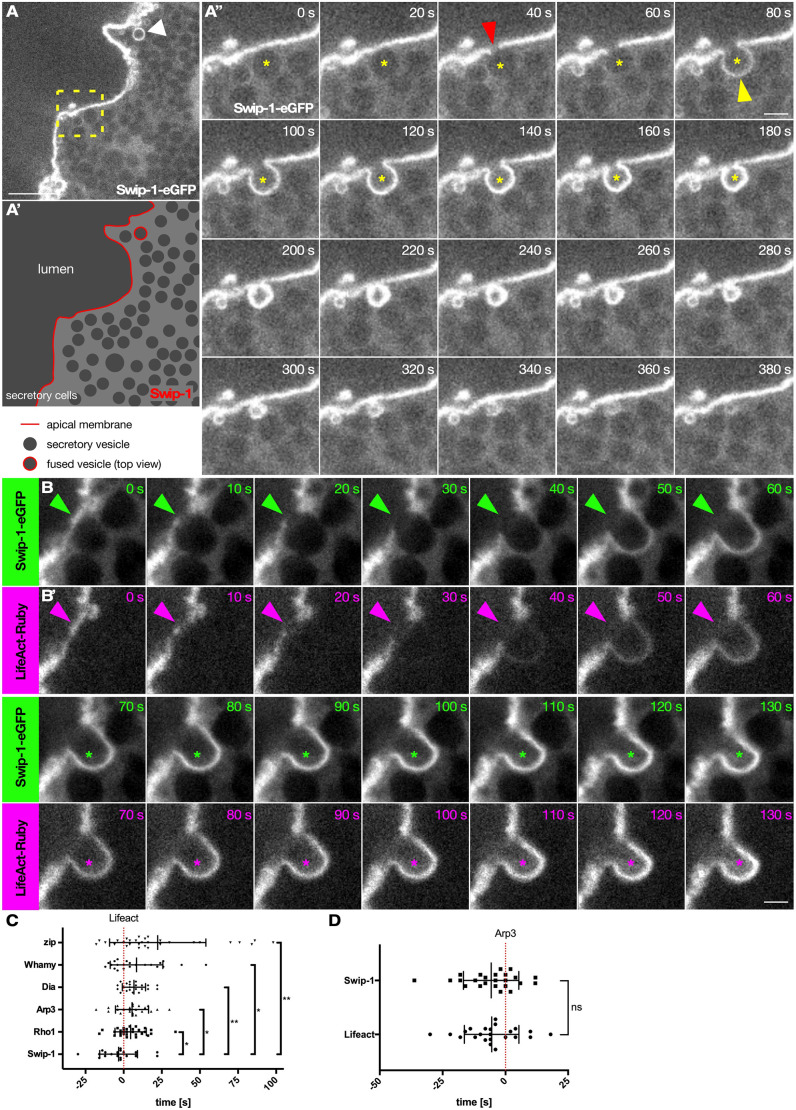
**Swip-1 is recruited to the membrane of fusing vesicle simultaneously with F-actin.** (A) Frame of spinning disk microcopy video of Swip-1-eGFP-expressing salivary gland. A secreting vesicle from top view is indicated by a white arrowhead. Scale bar: 25 µm. (A′) Schematic description of A. (A″) Frames of boxed area in A. Secreting vesicle is indicated by yellow asterisks. Red arrowhead shows clearing of Swip-1-eGFP at the apical membrane; yellow arrowhead indicates the subsequent coating of the vesicle with Swip-1-eGFP. Images were taken every 20 s. Scale bar: 5 µm. (B,B′) Detailed frames of spinning disk microscopy of a Swip-1-eGFP (green)- and LifeAct-Ruby (magenta)-expressing salivary gland. Green and magenta arrowheads indicate clearing at the apical membrane of the respective protein for fusion pore formation. Secreting vesicle is indicated by green and magenta asterisks. Images were taken every 10 s. Scale bar: 5 µm. Images are representative of at least three experiments. (C) Quantification of protein recruitment in relation to LifeAct (red dotted line, *t*=0). Fluorescence intensity was measured every 2 s. Swip-1, −3.31±12.37s, *n*=20 vesicles from seven salivary glands; Rho1, 4.55±10.25s, *n*=29 vesicles from eight salivary glands and three independent crosses; Arp3, 5.48±10.87s, *n*=23 vesicles from seven salivary glands; Dia, 6.73±7.60s, *n*=22 vesicles from six salivary glands and three independent crosses; Whamy, 8.45±17.25s, *n*=22 vesicles from seven salivary glands; Zip, 22.33±31.58s, *n*=30 vesicles from nine salivary glands and three independent crosses. Bars represent mean±s.d. (D) Quantification of LifeAct and Swip-1 recruitment in relation to Arp3 (red dotted line, *t*=0). Fluorescence intensity was measured every 2 s. LifeAct and Swip-1 are both detected at the vesicle membrane at the same time in relation to Arp3 detection. LifeAct, −5.48±10.87s, *n*=23 vesicles from seven salivary glands; Swip-1, −5.68±11.04s, *n*=24 vesicles from six salivary glands. Bars represent mean±s.d. ns, not significant (*P*>0.12), *P=0.033, **P=0.002 (Mann–Whitney test).

Western blot analysis and immunostainings confirmed prominent expression of Swip-1 in wild-type salivary glands from third-instar larvae (Fig. 1B,C). Before secretion, salivary gland cells became packed with numerous glue granules with a relatively homogenous diameter of ∼4–5 µm (15 µm^2^ in size), leaving little space for the cytoplasm (Fig. S1B,C). Endogenous Swip-1 protein mainly localized in the cytoplasm that forms a reticulated pattern around large glue granules (Fig. S1B″). In secretory salivary gland cells, however, endogenous Swip-1 became dramatically enriched at fused granules and colocalized with the F-actin that forms a coat on secretory vesicles as one of the early events in the secretory process (Fig. 1C; Rousso et al., 2016; Tran et al., 2015). A complete loss of immunostaining in mutant salivary glands confirmed the high specificity of the anti-Swip-1 antibody (Fig. 1D,D″). Similarly, an eGFP-tagged Swip-1 protein expressed by the *srp*-Gal4 driver selectively marked fused secretory vesicles colocalizing with F-actin (Fig. 1E,F). Live imaging of eGFP-tagged Swip-1 highlighted its dynamic localization in the early secretory process (Fig. 2A,A″; Movie 1). Swip-1 first localized along the apical membrane, became cleared at the point of vesicle fusion and formed a coat on the vesicle only after fusion to the apical membrane, as similarly reported for F-actin (Fig. 2A″; Movie 1). Live imaging of Swip-1-eGFP together with LifeAct-Ruby further revealed that Swip-1 and F-actin colocalized on secretory vesicles (Fig. 2B; Movie 2). As shown in Fig. 2C, the average time for the detection of Swip-1 in relation to LifeAct was −3.31±12.37 s (mean±s.d.). Using Arp3 recruitment as a reference, Swip-1 and LifeAct appeared simultaneously on vesicles (Fig. 2D). Thus, Swip-1 and F-actin were seen on granules before Arp3 recruitment as previously measured (Fig. 2C,D; Tran et al., 2015). Additional time-lapse co-labeling analyses using LifeAct combined with the Rho-GTP biosensor Anillin-RBD-eGFP (Movie 3; Munjal et al., 2015), Arp3-eGFP (Movie 4), Dia-eGFP (Movie 5) (Schmidt et al., 2021), the WASP-like protein Whamy (Movie 6) (Brinkmann et al., 2016) and the non-muscle myosin II marker (Zip-eGFP; Movie 7) further confirmed their later, sequential recruitment to the membrane of fused vesicles compared to Swip-1 (Fig. 2C). The average time differences for the detection of F-actin relative to Whamy and to myosin II were 8.45±17.25[Supplementary-material sup1]s and 22.33±31.58[Supplementary-material sup1]s, respectively (Fig. 2D).

### Loss of Swip-1 function impairs salivary gland secretion

**Fig. 3.**
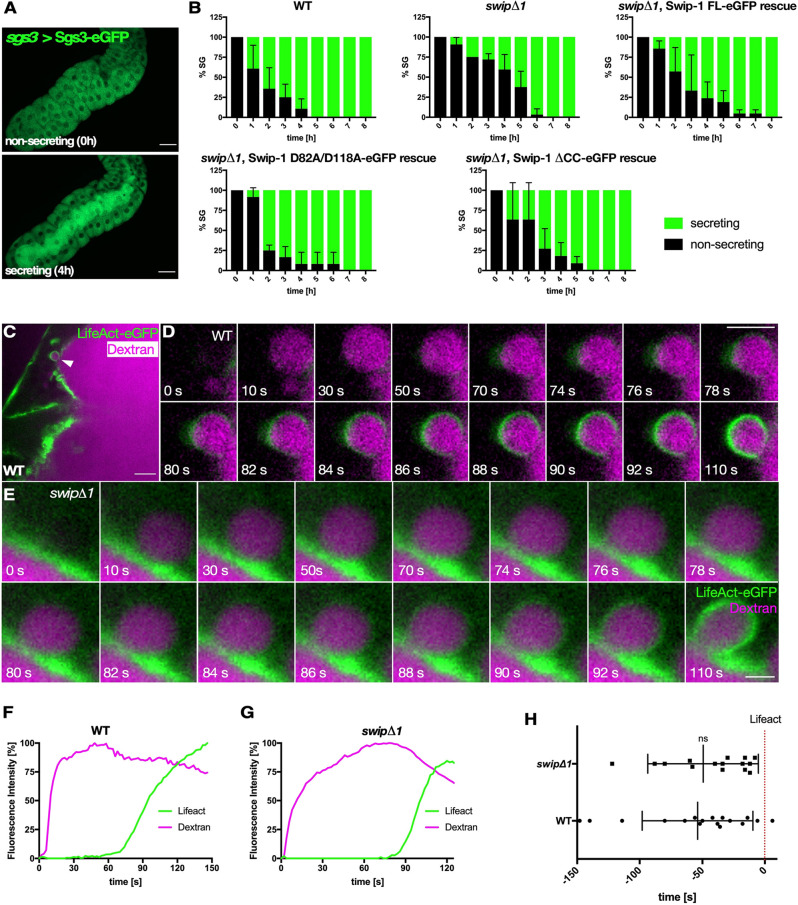
**Swip-1 is not required for fusion pore formation but rather for cargo expulsion.** (A) Salivary glands expressing Sgs3-eGFP used to determine secretion status. Secretory salivary glands display a bright green lumen (bottom). Scale bars: 100 µm. (B) Quantification of exocytosis of wild-type, *swip-1* mutant and rescued salivary glands. Exocytosis was induced by addition of 1 mM 20E, and salivary glands were assessed for secretion for 8 h with a spinning disk microscope. Apoptotic and non-secreting (unprimed) salivary glands were discarded from analysis. Green bars indicate percentage of secreting salivary glands (SG); error bars indicate s.d. Wild type, 28 salivary glands; *swip*Δ1, 32 salivary glands; Swip-1-eGFP rescue, 21 salivary glands from four independent crosses; Swip-1-D82A/D118A-eGFP rescue, 13 salivary glands from four independent crosses; Swip-1-ΔCC-eGFP rescue, 22 salivary glands from five independent crosses. Statistical analysis using the Mantel–Cox test revealed a significant difference only for *swip*Δ1 compared to wild type (*P*<0.001). (C) Frame of spinning disk microscopy video of salivary gland expressing LifeAct-eGFP (green) infused with Dextran (magenta). Scale bar: 10 µm. (D) Detailed frames of fusing vesicle in wild type. Scale bar: 5 µm. (E) Detailed frames of fusing vesicle in *swip-1* mutant. Images were taken every 2 s. Scale bar: 10 µm. Images are representative of at least three experiments. (F,G) Representative graphs of LifeAct recruitment to the vesicle membrane and time of dextran entry in wild type and *swip-1* mutant. (H) Quantification of time of Dextran entry in relation to LifeAct (red dotted line, *t*=0). There is no significant difference in time of Dextran entry between wild-type and *swip-1* mutant salivary glands. Wild type, −53.76±44.18s, *n*=17 vesicles from five salivary glands; *swip*Δ1, 49.00±43.14s, *n*=16 vesicles from five salivary glands. Error bars indicate s.d. ns, not significant (*P*=0.12) (Mann–Whitney test).

To further investigate whether Swip-1 function is required for regulated exocytosis, we next analyzed *swip-1* null mutants, which have recently been described (Lehne et al., 2022). To evaluate exocytosis of salivary gland cells quantitatively, we first analyzed the secretion of the GFP-fused glue protein (Sgs3-GFP) as a native cargo under the control of the endogenous *sgs3* promotor (Biyasheva et al., 2001). Before the onset of secretion, Sgs3-GFP was exclusively detected in salivary gland cells, whereas in secreting glands increasing levels of the fluorescent marker were released into the lumen within the first 4 h after 20E treatment (Fig. 3A, quantification in B). Compared to wild type, *swip-1* null mutant salivary glands showed a striking delayed secretion with most glands secreting 6 h post induction (Fig. 3B). Impaired secretion of *swip-1* mutant salivary glands was fully rescued by re-expression of a full-length swip-1 transgene (Fig. 3B). Interestingly, Ca^2+^ levels were elevated after 2 h of 20E treatment (Biyasheva et al., 2001), prompting us to test whether the function of Swip-1 depends on its Ca^2+^-binding ability. Remarkedly, we found that re-expression of Swip-1 protein deficient for Ca^2+^ binding (Swip-1-D82A/D118A) as well as a deletion construct lacking the coiled-coil domain (Swip-1-ΔCC) could substantially rescue impaired mutant secretion (Fig. 3B). Taken together, these data indicate that appropriate salivary gland secretion requires Swip-1 but not its functional Ca^2+^ binding or dimerization.

### Swip-1 function is not required for fusion pore formation, but rather for cargo expulsion of secreting vesicles

Swip-1 has previously been found to accumulate transiently in the foci of fusion-competent myoblasts in *Drosophila*, suggesting a possible role for Swip-1 in the breakdown of the prefusion complex during myoblast fusion (Hornbruch-Freitag et al., 2011). To assess a possible impact of Swip-1 on the formation of the fusion pore of secreting vesicles, we analyzed the time between fusion pore formation and actin recruitment to the secretory vesicles by infusing a low-molecular mass fluorescent dextran (10 kDa) into the lumens of salivary glands expressing LifeAct-GFP (Tran et al., 2015). Fluorescent dextran entered the vesicle immediately upon fusion pore formation before an F-actin coat marked by LifeAct-GFP was detected on vesicle membranes (Fig. 3D,E; Movie 8). Comparative analysis of wild-type and mutant salivary gland cells revealed no significant differences in the time between dextran diffusion into the vesicles and F-actin coat formation (Fig. 3F,G; Movie 8). The average time differences for the detection of dextran relative to F-actin in wild-type and mutant cells were 53.76±44.18 s and 49.00±43.14 s, respectively (Fig. 3H). Thus, these data imply that impaired secretion observed in mutant salivary glands is not accompanied by defective vesicle fusion pore formation.

**Fig. 4.**
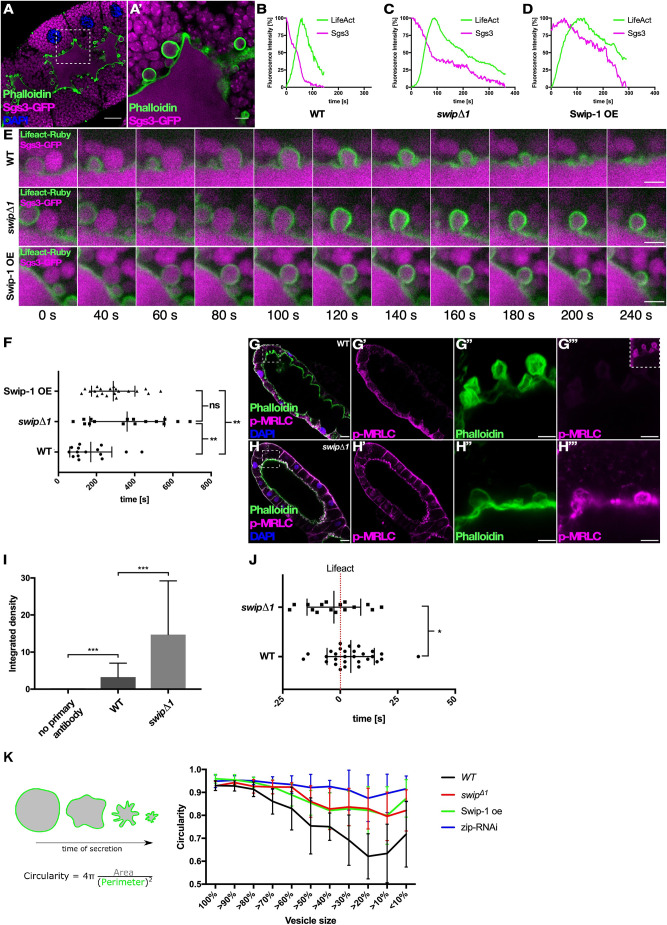
**Swip-1 promotes effective exocytosis of glue granules in *Drosophila* salivary glands.** (A) Confocal image of fixed Sgs3-GFP (magenta)-expressing salivary gland stained for F-actin (green) and DAPI (blue). Scale bar: 25 µm. (A′) Detailed view of boxed area in A. Vesicles are filled with glue protein and fused vesicles coated with F-actin. Scale bar: 5 µm. (B–D) Representative graphs of LifeAct recruitment and Sgs3 expulsion in wild-type (B), *swip-1* mutant (C) and Swip-1-overexpressing (Swip-1 OE; D) salivary glands. Fluorescence intensity was measured every 2 s. (E) Time series images of each genotype from representative graphs in B–D. Scale bars: 5 µm. (F) Quantification of expulsion duration defined as time from actin coat formation to minimal Sgs3-GFP detection. Cargo expulsion is significantly delayed in both *swip-1* mutant and Swip-1-overexpressing salivary glands. Bars represent mean±s.d. Wild type, 170.40±110.15s, *n*=15 vesicles from four salivary glands; *swip*Δ1, 360.88±195.91s, *n*=16 vesicles from four salivary glands; Swip-1 overexpression, 288.00±113.36s, *n*=20 vesicles from five salivary glands and two independent crosses. (G–G‴) Confocal images of a fixed wild-type secreting salivary gland from larvae stained for F-actin (green), active myosin [anti-phospho-myosin light chain antibody (p-MRLC), magenta] and DAPI (blue). (G′) Active myosin staining. (G″,G‴) Detailed views of boxed area in G, showing F-actin (G″) and active myosin (G‴) around fused vesicles. (H–H‴) Confocal images of a fixed *swip-1* mutant secretory salivary gland from larvae stained for F-actin (green), active myosin (magenta) and DAPI (blue). (H′) Active myosin staining. (H″,H‴) Detailed view of boxed area in H, showing F-actin (H″) and active myosin (H‴) around fused vesicles. Images of G and H were taken with same excitation laser intensity and adjusted to the same gray-level values. Scale bars: 25 µm (G,H) and 5 µm (G″,G‴,H″,H‴). Images are representative of at least three experiments. (I) Quantification of fluorescence intensity as integrated density of single vesicles (sum of the values of the pixels in the selection) from three independent stainings. Wild-type salivary glands only stained with secondary antibody served as negative control (background fluorescence). Outliers of analysis were removed with ROUT method (Q=1%). Bars represent mean±s.d. Negative control, 0.04±0.05, *n*=23 vesicles from six salivary glands; wild type, 3.24±3.76, *n*=41 vesicles from ten salivary glands; *swip*Δ1, 14.75±14.46, *n*=40 vesicles from ten salivary glands. (J) Quantification of the time of Rho-GTP biosensor detection in relation to LifeAct (red dotted line, *t*=0) at the vesicle membrane in wild type (4.55±10.25s, *n*=29 vesicles from eight salivary glands and three independent crosses) and *swip-1* mutant (−2.75±11.63s, *n*=16 vesicles from five salivary glands and three independent crosses). (L) Left: scheme for analysis of vesicle crumpling during exocytosis. Single secreting vesicles of salivary glands expressing LifeAct-GFP were followed and manually traced for each time point (10 s interval). Circularity of the vesicle was calculated using Shape Descriptors in ImageJ. Right: area and circularity of the vesicles was measured over time, and the circularity was plotted as function of the normalized vesicle size to account for variable secretion durations. Wild-type vesicles (black line) show a reduced circularity especially in the last third of secretion. *zip* RNAi-expressing salivary glands (blue line) have almost no visible membrane crumpling of fusing vesicles. *swip-1* mutant and Swip-1-overexpressing salivary glands (red and green line, respectively) show reduced crumpling but less severe than that in *zip* RNAi-expressing salivary glands. All genotypes, *n*=20 vesicles from four salivary glands. ns, not significant (*P*=0.12), *P=0.033, **P=0.002, ***P<0.001 (Mann–Whitney test).

Intrigued by the established F-actin-binding activity of Swip-1 (Lehne et al., 2022), we further investigated whether Swip-1 is involved in late events in the secretory process, such as cargo expulsion, which requires a dense contractile actin network. To better monitor the complete process of apical vesicle secretion, we imaged salivary glands co-expressing Sgs3-GFP and LifeAct-Ruby, which allowed us to better visualize the shape and dynamics of vesicles during secretion (Fig. 4A,A′,B,E; Movie 9). Co-labeling with LifeAct-Ruby revealed that granules properly fused with the apical plasma membrane in *swip-1* mutant salivary gland cells, but often failed to collapse and secrete their contents into the lumen (Fig. 3C,E; Movie 10). Quantification further confirmed that the duration of size reduction and thereby cargo release was significantly prolonged in *swip-1* mutant cells, more than twice long as for wild type (wild type, 170.40±110.15 s; *swip*Δ1, 360.88±195.91 s; Fig. 4F). Interestingly, the overexpression of Swip-1 resulted in a similar prolonged cargo release (Fig. 4C), suggesting that the increased Swip-1 protein level interferes with its function, as found for many proteins that function as part of multiprotein complexes (Fig. 4D–F; Movie 11).

Previous studies revealed that individual wild-type secretory vesicles undergo a slight expansion directly after fusion with the apical plasma membrane, likely due to hydration-related expansion of mucins creating mechanical forces that will be counter-balanced by the branched actomyosin vesicle coat (Tran et al., 2015). Active myosin II (detected by an anti-phospho-myosin light chain antibody) was still recruited to fused vesicles in *swip*Δ1 mutants (Fig. 4G,H). By contrast, the level of active myosin II was strongly increased by 4.5-fold compared to that in wild type (Fig. 4I). Thus, increased levels of active myosin might reduce myosin dynamics and further increase vesicle stiffness, resulting in inefficient vesicle compression and secretion, as observed in *swip*Δ1 mutant salivary glands. Consistently, we observed prominent differences in Sqh-GFP recruitment between rescued *swip-1* mutant cells and cells overexpressing Swip-1 (Fig. S1D). Sqh-GFP localizes significantly earlier in vesicles in rescued *swip*Δ1 mutants than in vesicles in cells overexpressing Swip-1 (Fig. S1D). Comparative co-labeling analyses further revealed that the Rho-GTP biosensor (Anillin-RBD-eGFP) appeared significantly earlier on vesicles in *swip*Δ1 mutants compared to wild type (Fig. 4J), suggesting that Swip-1 might affect Rho signaling as previously observed in cultured B16F10 melanoma cells (Huh et al., 2015), which in turn affects actomyosin activity.

### Loss of Swip-1 function affects actomyosin-mediated vesicular membrane crumpling

A recent study revealed that the vesicular membrane becomes progressively folded by contraction of the actomyosin meshwork, which squeezes the content out of the vesicle while retaining and sequestering its membrane, giving it a crumpled appearance (Kamalesh et al., 2021). Thus, changes in actomyosin assembly and contractility on the vesicle membrane should affect membrane crumpling and thereby cargo release. To further test whether membrane crumpling of secreting vesicles is indeed affected in *swip*Δ1 mutants, we measured the circularity of secreting vesicles over time, normalized to vesicle size at variable stages of secretion (scheme in Fig. 4K). Wild-type vesicles undergo progressive membrane folding with significantly reduced circularity during secretion (Fig. 4K). By contrast, upon RNA interference (RNAi)-mediated suppression of the myosin II heavy chain (*zip* RNAi), vesicles retained their initial spherical shape, and almost no visible membrane crumpling was found in the last third of secretion (Fig. 4K) (Kamalesh et al., 2021). Interestingly, both *swip-1* mutant and Swip-1-overexpressing salivary glands showed reduced membrane crumpling, although less severe than that in *zip* RNAi-expressing salivary glands (Fig. 4K). Taken together, our studies identified a novel function of Swip-1 in regulating actomyosin activity in secretory cargo expulsion during regulated exocytosis.

## DISCUSSION

Swip-1 has been described as a conserved Ca^2+^-regulated actin-binding protein, which is broadly expressed in different cells and tissues across species and involved in diverse cellular functions, including immune defense, cell migration and endocytosis (Huh et al., 2013, 2015; Kwon et al., 2013; Lehne et al., 2022; Moreno-Layseca et al., 2021; Tu et al., 2018). However, a role of Swip-1 in exocytosis has not yet been reported.

Exocytosis is an actin-driven sequential process by which proteins are delivered in membranous secretory vesicles to the extracellular space, either constitutively or upon stimulation, in a process called regulated exocytosis. Synthesis of salivary gland glue mucins is stimulated by a short pulse of the steroid hormone 20E in early third-instar larvae (Biyasheva et al., 2001). Glue proteins are initially stored in large secretory vesicles and then released into the lumen upon a second pulse of 20E at the end of the third larval instar. Cargo release proceeds in sequential steps that depend on coordinated assembly and organization of an actin cytoskeletal network (Tran and Ten Hagen, 2017). Secretion begins with the clearance of F-actin at the apical plasma membrane, followed by fusion pore formation and subsequent formation of an F-actin coat surrounding fused secretory vesicles (Tran and Ten Hagen, 2017). A model has been proposed in which two central actin nucleators, Dia and the Arp2/3 complex, are sequentially recruited to fusing vesicles. First, Dia forms an initial linear actin filament coat. Second, Arp2/3 and its activators such as WASP, are subsequently recruited to promote the formation of branched actin coat structures. Together with myosin II, this actin meshwork provides the mechanical forces needed to fold the membrane and thereby squeezes the content into the apical lumen (Rousso et al., 2016; Tran et al., 2015). Our data further support this two-step model, but also indicate a central role of the Rho-GTPase in the initial F-actin coat formation. Our localization data suggest that Rho precedes Dia recruitment. Rho promotes Dia-mediated actin nucleation (Bogdan et al., 2013; Kuhn and Geyer, 2014; Spiering and Hodgson, 2011), but also promotes actomyosin contractility through activation of the Rho-dependent kinase Rok (Amano et al., 1996; Hodge and Ridley, 2016). Interestingly, we found that Swip-1 is recruited simultaneously with F-actin to fused secretory vesicles, suggesting an early function in initial F-actin coat formation. Previous studies suggested that Swip-1 binds to F-actin through multiple actin-binding sites, but the actin-binding sites on Swip-1 have not been mapped exactly (Kwon et al., 2013; Park et al., 2016). Deletion of the first EF-hand domain (EF1) renders Swip-1 unable to bind actin, highlighting a central actin-binding site within the EF1 (Moreno-Layseca et al., 2021). Swip-1 can also act on Rho-GTPase signaling. RNAi-mediated suppression of Swip-1 increases RhoA activity, whereas Swip-1 overexpression reduces RhoA activity (Huh et al., 2015). Thus, independent of its Ca^2+^-dependent cross-linking activity, Swip-1 might play a conserved role in regulating RhoA activity or localization in salivary gland secretion. Supporting this notion, we found that loss of Swip-1 results in a significant premature recruitment of Rho1 (the single RhoA homolog in *Drosophila*), which explains increased actomyosin activity in *swip*Δ1 mutants. This Ca^2+^-independent role of Swip-1 in regulating Rho might explain why neither Ca^2+^ binding nor dimerization of Swip-1 is required for proper secretion. Thus, we hypothesized that Swip-1 contributes to the recruitment of Rho-GTPase regulating actomyosin activity to drive proper vesicle membrane crumpling and expulsion of cargo.

In summary, we identified a novel function of the conserved cross-linker Swip-1 in regulated exocytosis. The function of Swip-1 in secretory cells could be conserved between flies and humans. Human Swip-1 (also known as EFHD2) has been found in a number of pathophysiological conditions and has been proposed as a potential biomarker for chronic diseases, including diseases associated with synaptic dysfunction (Kogias et al., 2019; Mielenz and Gunn-Moore, 2016; Thylur et al., 2018). However, the role of Swip-1 in pathogenesis and the underlying molecular mechanism are not well understood. Thus, future studies are expected to dissect the pathophysiological role of Swip-1 in more specialized types of exocytosis, including synaptic transmission.

## MATERIALS AND METHODS

### *Drosophila* genetics

Fly husbandry and crossing were carried out according to standard methods. All crosses were performed at 29°C. The following fly stocks were obtained from the Bloomington Drosophila Stock Center: w[1118] (BL3605), y[1] w[*]; Mi{Trojan-Gal4.0}ptc[MI02003-TG4.0] (BL67438), w[1118]; P{w[+mC]=Sgs3-GAL4.PD}TP1 (BL6870), P{w[+mC]=UAS-LifeAct.GFP.W}3 (BL57326), y[1] w[*]; P{y[+t*] w[+mC]=UAS-LifeAct-Ruby}VIE-19A (BL35545), w[*]; P{w[+mC]=Sgs3-GFP}3 (BL5885), w[*]; P{w[+mC]=PTT-GC}Zip[CC01626]/SM6a (BL51564), w[*]; P{w[+mC]=sqh-mCherry.M}3 (BL59024). The following RNAi stocks were obtained from the Vienna Drosophila Resource Center: w[1118]; P{GD7047}v31308 (*swip-1* RNAi), w[1118]; P{GD1566}v7819 (*zip* RNAi) and w1118; P{GD14716}v29944 (*arp2* RNAi). The y,v; srp-Gal4 stock was a kind gift from Daria Siekhaus (Institute of Science and Technology Austria, Klosterneuburg, Austria). The Dia-GFP stock was a kind gift from Jörg Grosshans (Philipps-University Marburg, Germany) (Schmidt et al., 2021). The UASp-GFP-Arp3 stocks (referred to as UAS-Arp3-eGFP) were a kind gift from Gaia Tavosanis (Deutsches Zentrum für Neurodegenerative Erkrankungen, Bonn, Germany). The w[1118]; Ubi-Anillin-RBD-eGFP stocks were a kind gift from Thomas Lecuit (Centre national de la recherche scientifique, Marseille, France). Transgenic UAST-Whamy-eGFP (Brinkmann et al., 2016), UAST-Swip-1, UAST-Swip-1-eGFP, UAST-eGFP-Swip-1, UAST-Swip-1-D82A/D118A-eGFP, UAST-Swip-1-ΔCC-eGFP and UAST-Swip-1-mScarlet-I flies were generated using ΦC31-mediated transgenesis: y[1] M{vas-int.Dm}ZH2A w[*]; M{3xP3-RFP.attP’}ZH-86Fb (BL24749) and y[1] M{vas-int.Dm}ZH2A w[*]; M{3xP3-RFP.attP’}ZH-68E (BL24485) (Bischof et al., 2007). The *swip*Δ1 mutant was generated by CRISPR/Cas9 of the following target sequence: 5′-GGGGTCTTCGAGAAGACCT-3′ (Lehne et al., 2022). Loss of the Swip-1 protein was confirmed by western blot analysis of His-Swip-1 (Pineda, Berlin). The following stocks were established for live imaging: BL67438 was recombined with BL35545 to obtain ptc-Gal4, UAS-LifeAct-Ruby and further established with UAS-Swip-1, UAS-Swip-1-eGFP, UAS-Arp3-eGFP, UAS-Whamy-eGFP or Sgs3-GFP. The stock was further recombined with *swip*Δ1 to obtain ptc-Gal4, UAS-LifeAct-Ruby, *swip*Δ1, and this stock was further established with Sgs3-GFP. BL67438 was recombined with UAS-eGFP-Arp3 to obtain ptc-Gal4, UAS-eGFP-Arp3, which was then established with UAS-Swip-1-mScarlet. srp-Gal4 was recombined with BL57326 to obtain srp-Gal4, UAS-LifeAct-GFP. All eGFP-tagged Swip-1 variants were recombined with srp-Gal4 and crossed in the *swip-1* mutant background to obtain srp-Gal4, UAST-Swip-1-eGFP and *swip*Δ1; srp-Gal4, UAST-Swip-1-eGFP/TM6B, *swip*Δ1; srp-Gal4, UAST-Swip-1-D82A/D118A-eGFP, TM6B and *swip*Δ1; srp-Gal4, UAST-Swip-1-ΔCC-eGFP. Lastly *swip*Δ1; Sgs3-GFP was established.

### Antibody generation

The rabbit anti-Swip-1 antibody was generated against the full-length *Drosophila* Swip-1 fused to a 6xHis-tag (pDEST17, Thermo Fisher Scientific). The 6xHis-Swip-1 fusion protein was expressed in *Escherichia coli* and purified with Ni-NTA resin (GE Healthcare). Rabbits were immunized with purified proteins by Pineda Antikörper-service (Berlin, Germany).

### Validation of Swip-1 knockout

Swip-1 null mutants were validated by isolating 20 salivary glands from third-instar wandering larvae, removing the fat body, squashing the glands in 15 µl 2× SDS sample buffer and incubating at 95°C for 10 min for SDS-PAGE. The following antibodies were used for western blot analysis: anti-Swip-1 (1:5000, purified from rabbit; Lehne et al., 2022), anti-actin AB-5 (1:5000, AB_2289199, BD Biosciences), goat anti-rabbit IgG (H+L)-HRP (1:5000, 31460, Thermo Fisher Scientific) and goat anti-mouse IgG (H+L)-HRP (1:5000, 31430, Thermo Fisher Scientific).

### Immunohistochemistry and fluorescence staining

For antibody staining, salivary glands of third-instar wandering larvae were isolated in 1× PBS, fixed in 4% formaldehyde in phosphate buffer pH 7.4, washed and blocked with 5 mg/ml bovine serum albumin in 50 mM Tris-HCl pH 7.4+0.5% Nonidet P-40 and the anti-Swip-1 antibody (1:5000 dilution) or the anti-phospho-Myosin Light Chain 2 (Ser19) antibody (1:20 dilution; 3671, Cell Signaling Technology) and incubated overnight. The primary antibody was visualized with polyclonal Alexa Fluor-568-conjugated goat-anti-rabbit antibody (1:1000 dilution; A11036, Invitrogen). F-actin was visualized using Alexa Fluor-488-conjugated Phalloidin (1:100 dilution; A12379, Invitrogen), and nuclei were visualized by DAPI staining (1 µg/ml; 62248, Thermo Fisher Scientific). For only staining of F-actin and nuclei, dissected glands were fixed in 4% paraformaldehyde in 1× PBS, washed once with 1× PBS+0.5% Triton X-100, washed three times with 1× PBS and stained with Alexa Fluor-488-conjugated Phalloidin (1:100 dilution; A12379, Invitrogen) and DAPI (1 µg/ml; 62248, Thermo Fisher Scientific). Glands were mounted in Fluoromount-G Mounting Medium (Invitrogen).

### Fluorescence microscopy

Confocal images were taken with a Leica TCS SP8 with an HC PL APO CS2 40×/1.4 NA oil objective. For quantification of anti-phospho-Myosin Light Chain 2 (Ser19) staining, a hybrid detector (Leica HyD) in photon counting mode was used. Single vesicles were traced in one *z*-plane using freehand selections in ImageJ, and the integrated density with s.d. was calculated. Larval salivary glands stained only with the secondary antibody served as negative control. Quantification of the cross-section area of packed vesicles of non-secreting larval salivary glands was performed according to Ma and Brill (2021). In short, Sgs3-GFP-expressing larval salivary glands were imaged using Leica Lightning Deconvolution software. Three cells of three glands for each genotype were scanned and analyzed in three *z*-planes with 3 µm distance to account for size variability throughout the gland. Cross-section area of single vesicles was determined by detection and measuring the surface in Imaris 9.3 (Bitplane). Surfaces with values below 4 µm^2^ were excluded from analysis.

### Live-cell imaging of larval salivary glands

Live imaging of larval salivary glands was performed as previously reported (Tran et al., 2015). In short, naturally secreting isolated glands were placed in a glass-bottom imaging dish, covered with a Isopore 0.1 µm PC membrane (Merck) and 50 µl Schneider's *Drosophila* medium. For long-term imaging, the imaging chamber was humidified with a wet tissue paper and sealed with parafilm to prevent evaporation. Dissected glands were imaged with a Zeiss CellObserver Z.1 with a Yokogawa CSU-X1 spinning disc scanning unit and an Axiocam MRm CCD camera (6.45 µm×6.45 µm). For additional Dextran imaging, dissected glands were incubated for 1 h with 200 µM Dextran-Alexa568 (molecular mass 10,000, Invitrogen) in Schneider's *Drosophila* medium, washed three times with medium and then placed on an imaging chamber as described above.

### Quantification of exocytosis

To quantify the onset of secretion, salivary glands of third-instar wandering larvae expressing Sgs3-GFP under its native promotor were placed in Schneider's *Drosophila* medium supplemented with 1 mM 20E (Sigma-Aldrich) to induce exocytosis and live imaged for 8 h as described above. Every hour, detection of GFP fluorescence in the lumen was assessed. Glands that were already secreting at the beginning of imaging and glands that became apoptotic before onset of secretion were excluded from analysis. Also, glands that did not start secretion after 8 h of ecdysone induction were discarded as these most likely had not been primed by the first endogenous ecdysone pulse. Ratios of non-secreting and secreting glands were calculated for every time point. For rescue experiments, transgenic Swip-1 constructs were expressed using the srp-Gal4 driver in the *swip-1* mutant background.

### Analysis of protein recruitment

Larval salivary glands were imaged every 2 s for 10–20 min. To compare time of recruitment to the vesicle membrane, measurement of percentage fluorescent intensity of two proteins of interest (e.g. LifeAct and Swip-1) was performed as previously described (Tran et al., 2015). In short, an oval region of interest was drawn around single fusing vesicles in a single *z*-plane. The ‘plot z-axis profile’ function in ImageJ was used to obtain fluorescence intensity values for each channel. Subsequent calculations of percentage fluorescence intensity and time difference for detection were carried out in Excel (Microsoft) according to Tran and colleagues (Tran et al., 2015). Start of protein recruitment to the vesicle membrane was defined as florescence intensity increase of ≤1% compared to the previous frame. Accordingly, measurement of time of cargo expulsion was determined by calculating the time difference between detection of LifeAct and minimal Sgs3 fluorescence in the region of interest. For data plotting, Sgs3 fluorescence at onset of actin coat formation was set to 100% and minimal detection of fluorescence at end of cargo expulsion (i.e. background fluorescence) was set to 0%.

### Analysis of membrane crumpling

Larval salivary glands expressing LifeAct were imaged every 10 s for 30 min. Single vesicles were followed and their membrane traced with ‘freehand selections’ in ImageJ. Circularity of the vesicle for every time point was calculated with ImageJ shape descriptors. Obtained values were normalized for the vesicle size (measured area), and mean values of circularity calculated for every vesicle size decile were calculated.

### Statistical analysis

Quantitative experiments were performed at least in four replicates to avoid any possible bias by environmental effects or unintentional error. Exact numbers of replicates and, where applicable, number of independent crosses are specified in the figure legends. Raw data were processed in Excel (Microsoft). Statistical analyses were performed using Prism 7 (GraphPad). Outliers of all recruitment calculations were identified using the ROUT method (Q=2%) and excluded from further statistical analysis. To evaluate statistical significance, unless otherwise stated, the Mann–Whitney test was used and the *P*-value (two-tailed) was obtained [*P*=0.12 (not significant), *P=0.033, **P=0.002, ***P<0.001].

## Supplementary Material

10.1242/joces.260366_sup1 Supplementary information
