# Supplementary material for: Swip-1 promotes exocytosis of glue granules in the exocrine Drosophila salivary gland
Source: J Cell Sci. 2023 Mar 6;136(6):jcs260366. doi: 10.1242/jcs.260366 (PMC10038153; doi:10.1242/jcs.260366)
Supplement: Supplementary information [file joces-136-260366-s1.pdf]

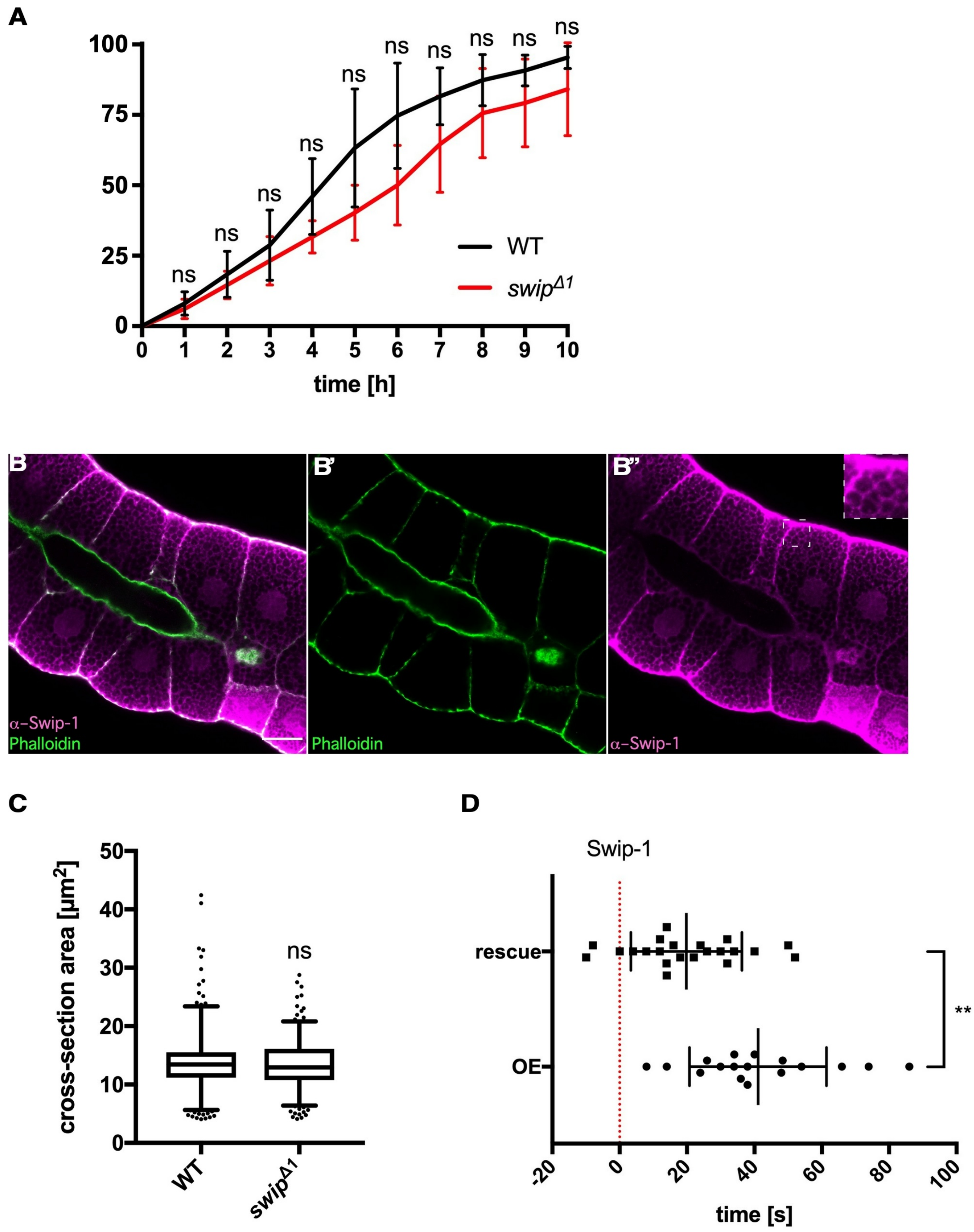

Fig. S1. Mutant *swip*-1 larvae show no developmental delay or reduced vesicle size in their salivary glands.

(**A**) To determine a developmental defect, wild-typic and *swip*-1 mutant wandering L3 larvae were collected in a humidified petri dish and kept on 25°C. The number of developed prepupae were counted every hour for 10h. There is no significant difference in wild type and swipΔ1 at any time point. Data points from 4 independent experiments. WT: n=83 and *swip*Δ1: n=69 larvae. (**B-B''**) Confocal images of a fixed wild-typic non-secreting salivary gland stained for F-actin (Phalloidin, green) and Swip-1 (magenta). Swip-1 is located in the cytoplasm and enriched at the plasma membrane (see inlet in B''). Scale bar 25μm. (**C**) Cross-section area of packed vesicles in non-secreting salivary glands. (D) Quantification of Sqh-mCherry recruitment relative to Swip-1-eGFP expression under the srp-Gal4 driver rescued swip-1 mutant and overexpression background. Myosin

Journal of Cell Science • Supplementary information

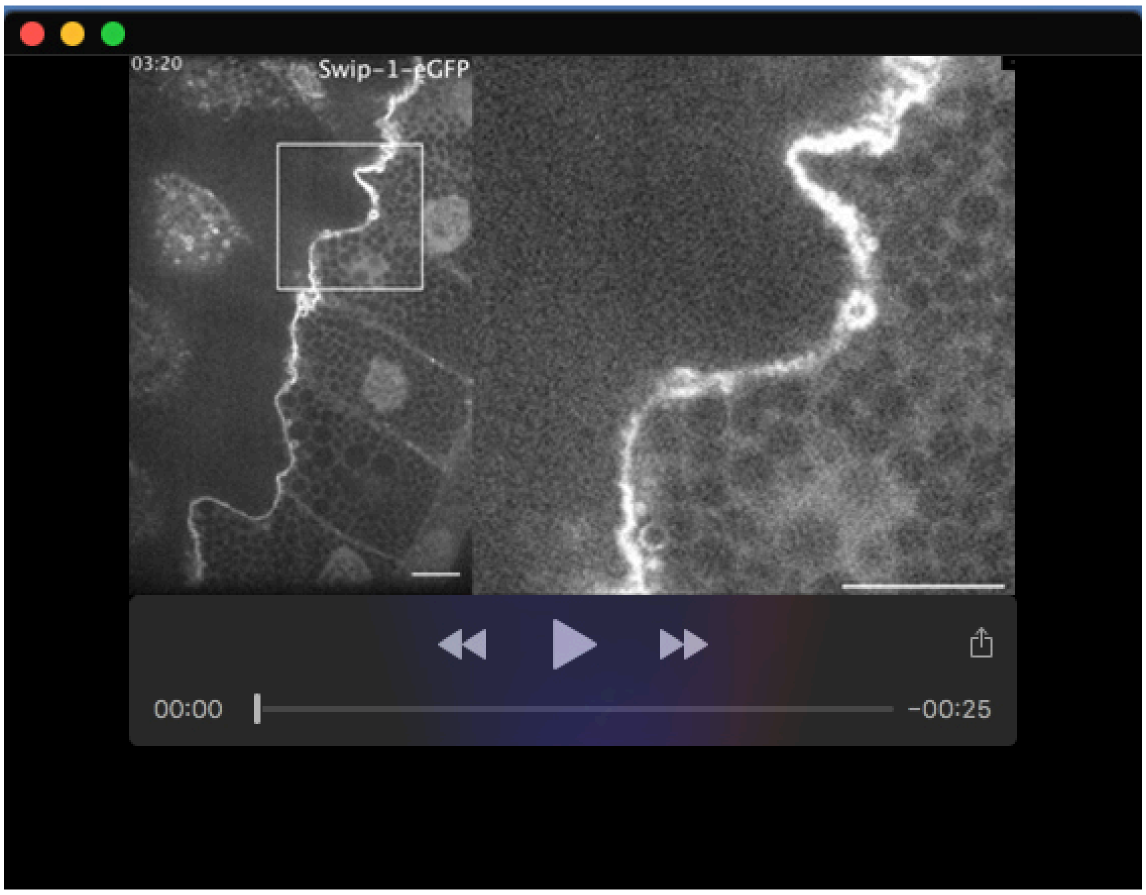

Movie 1. Time-lapse movie of *ex vivo* cultured salivary glands expressing UAS-Swip-1-eGFP under the srp-Gal4 driver. Right side shows the boxed area corresponding to the still image of Figure 2A. Scale bars 25µm.

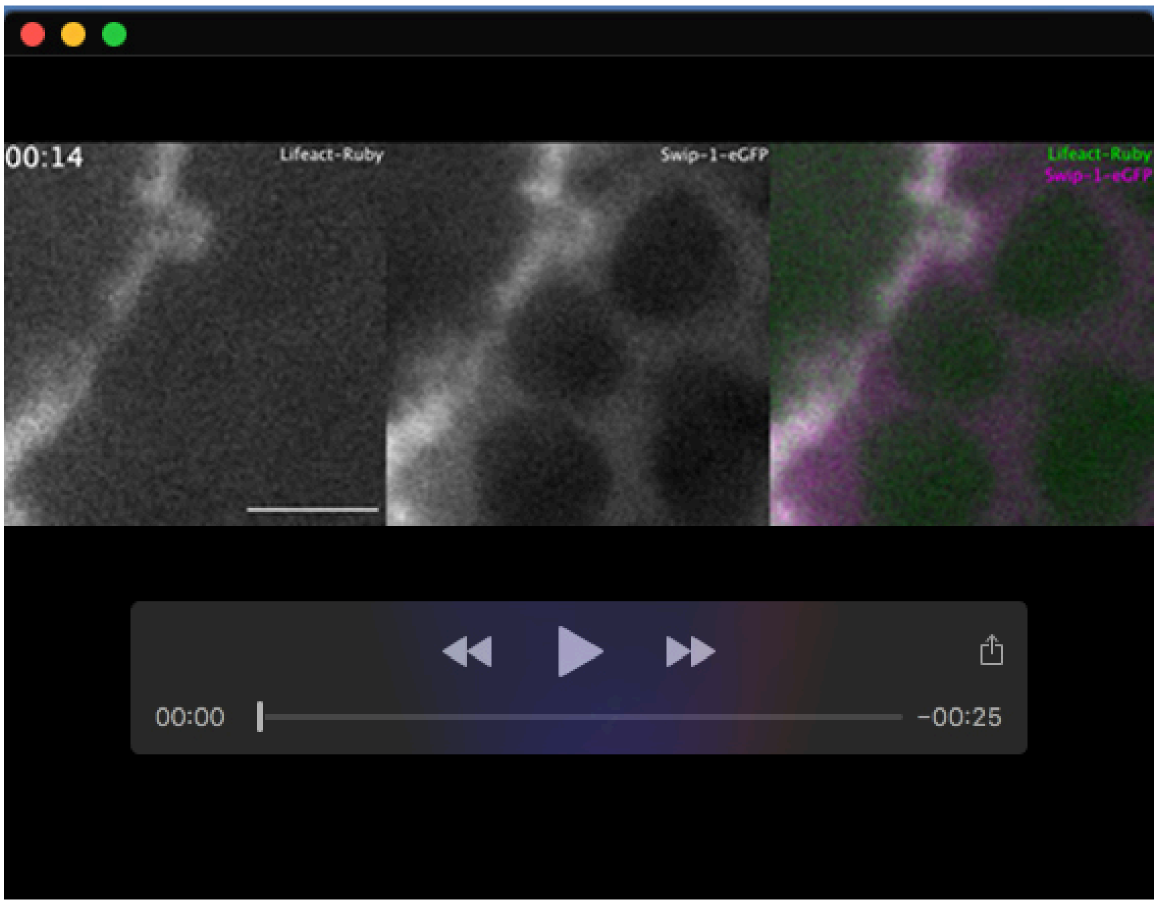

Movie 2. Representative time-lapse movie of *ex-vivo* cultured salivary glands co-expressing UAS-LifeAct-Ruby (green) and UAS-Swip-1-eGFP (magenta) under the ptc-Gal4 driver. Scale bar 5µm.

Journal of Cell Science • Supplementary information

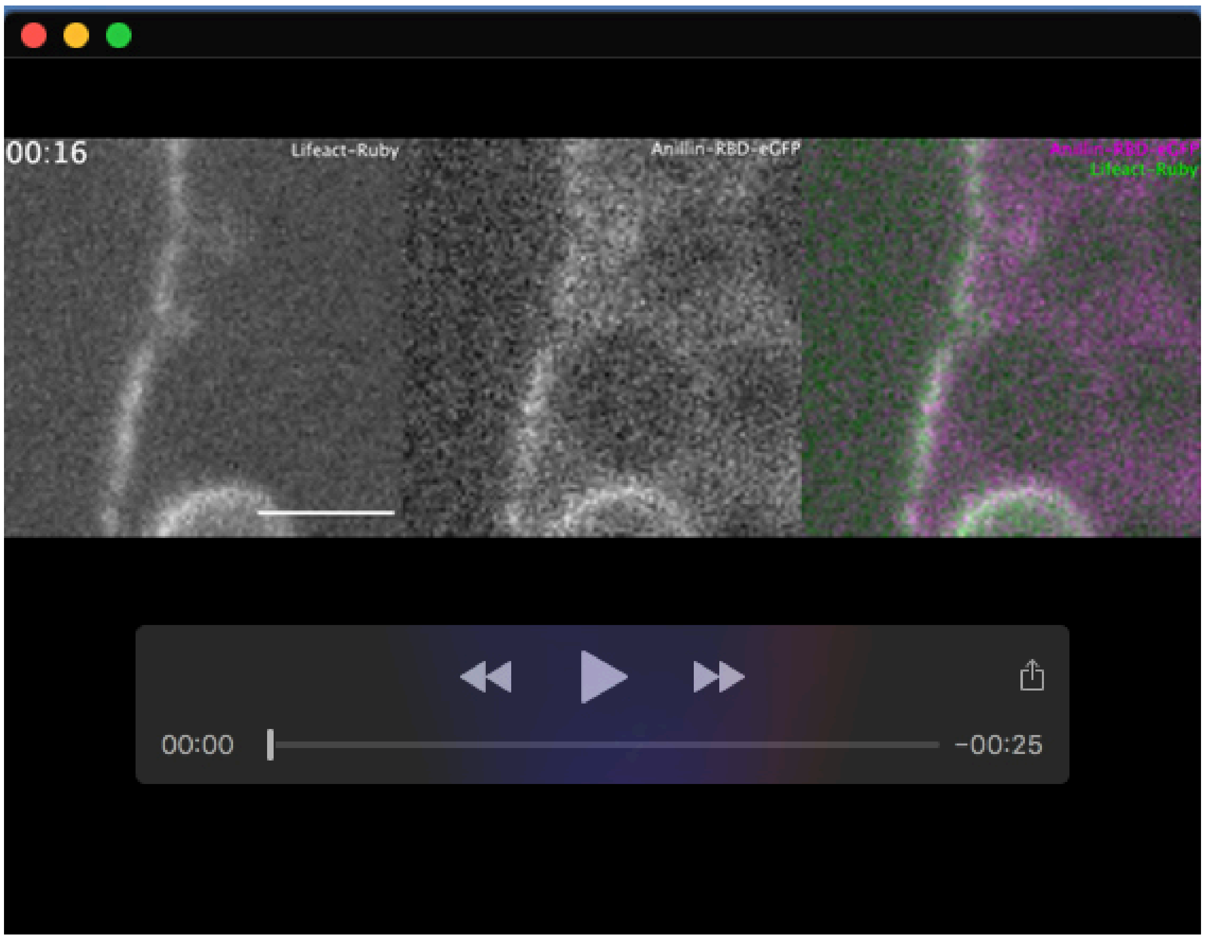

Movie 3. Representative time-lapse movie of *ex-vivo* cultured salivary glands co-expressing UAS-LifeAct-Ruby (green) under the ptc-Gal4 driver and the Rho-sensor Anillin-RBD-eGFP (magenta). Scale bar 5µm.

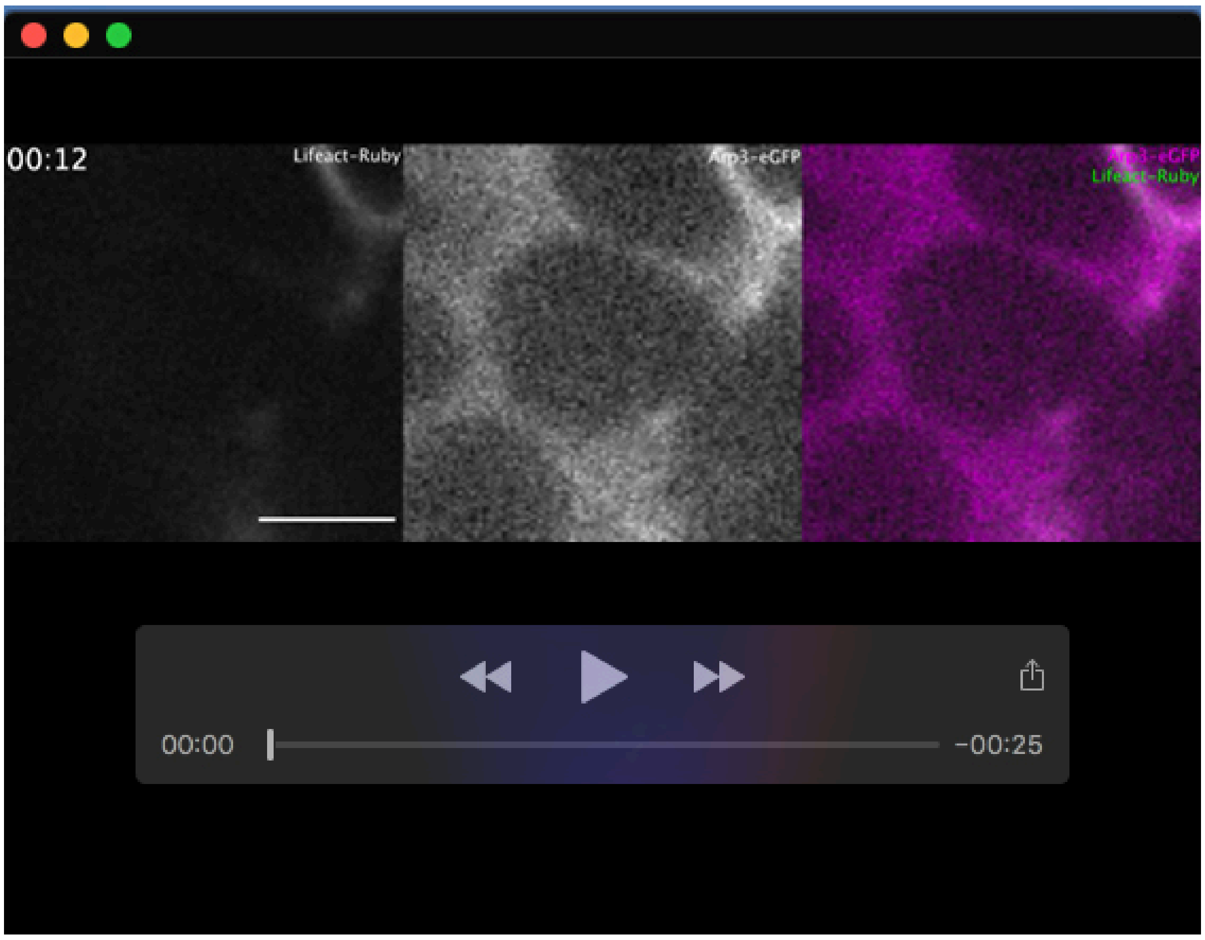

Movie 4. Representative time-lapse movie of *ex-vivo* cultured salivary glands co-expressing UAS-LifeAct-Ruby (green) and UAS-Arp3-eGFP (magenta) under the ptc-Gal4 driver. Scale bar 5µm.

Journal of Cell Science • Supplementary information

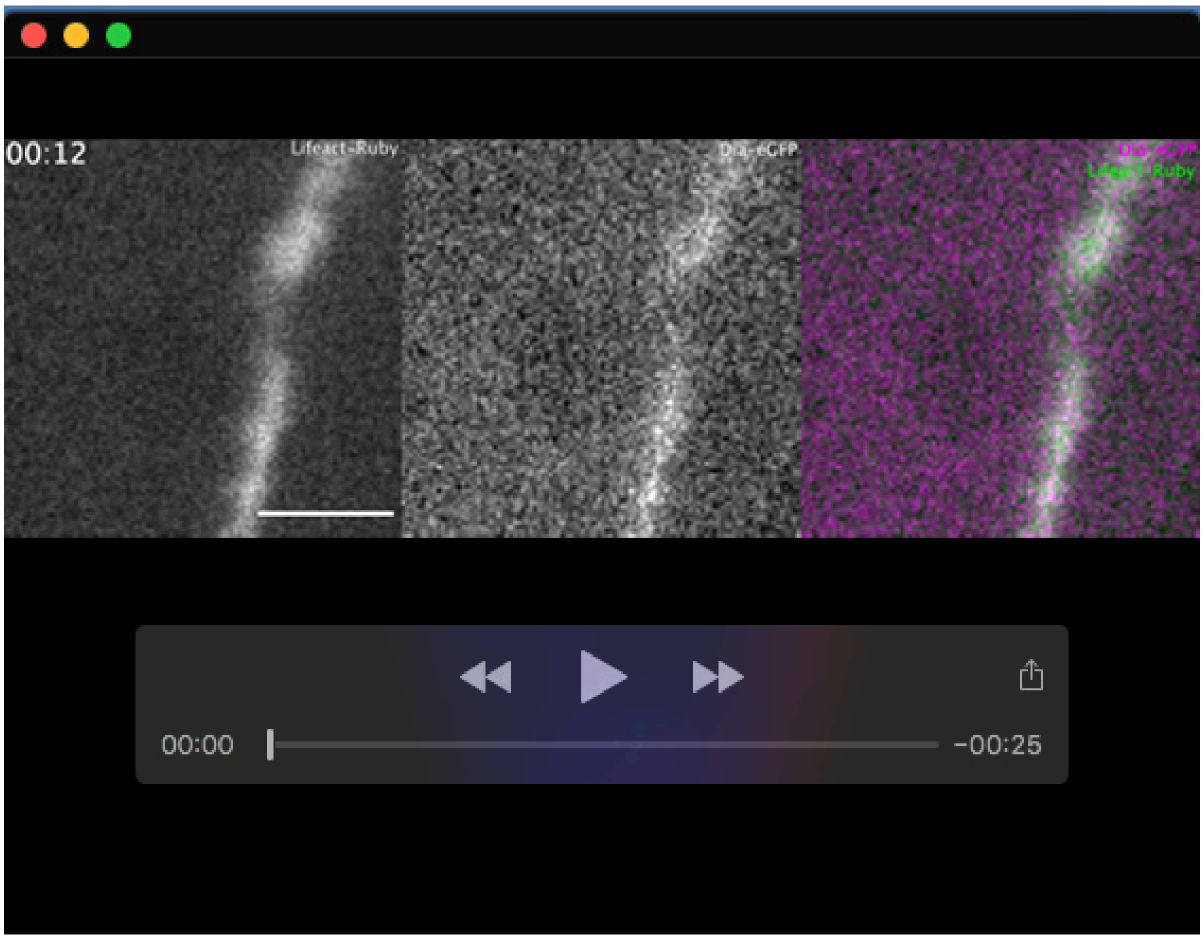

Movie 5. Representative time-lapse movie of *ex-vivo* cultured salivary glands co-expressing UAS-LifeAct-Ruby (green) under the ptc-Gal4 driver and Dia-eGFP (magenta). Scale bar 5µm.

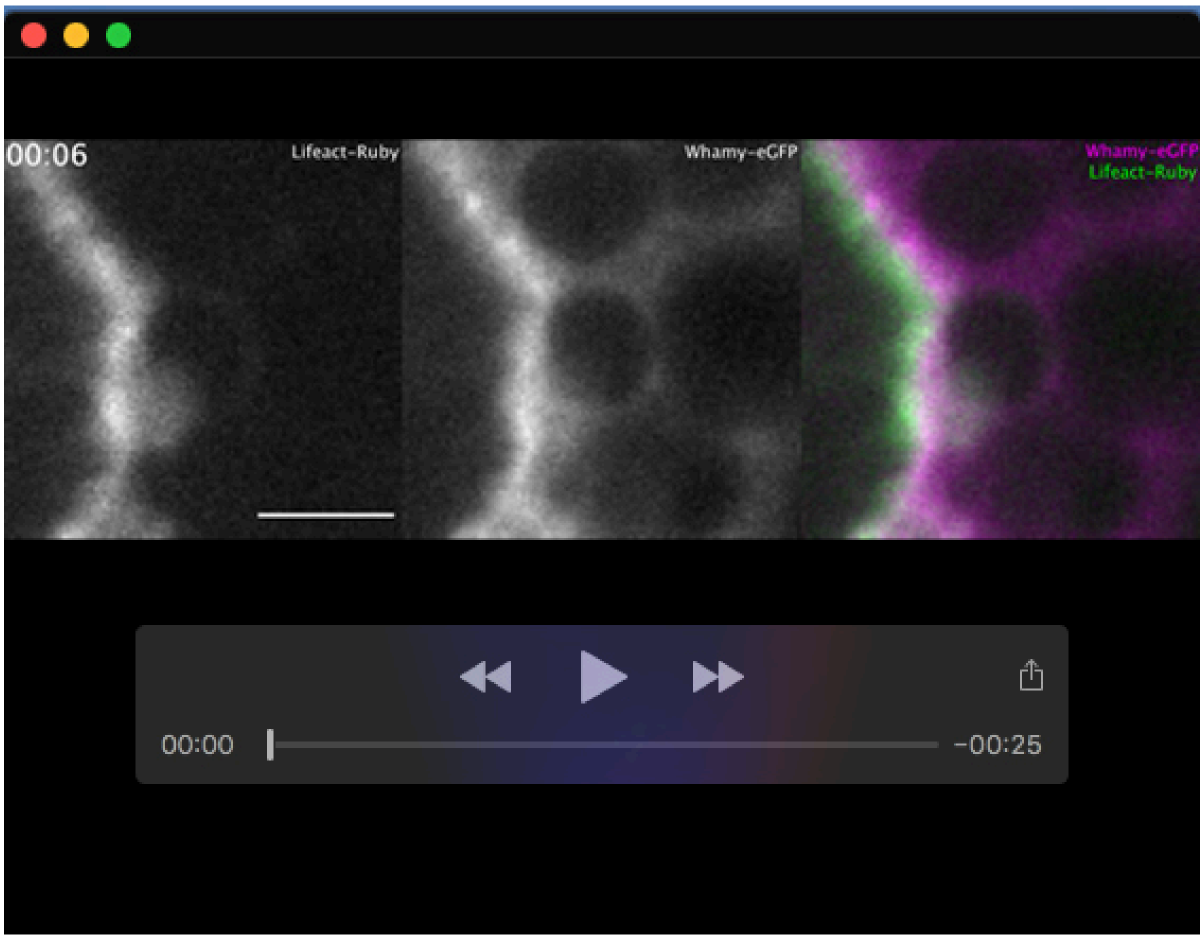

Movie 6. Representative time-lapse movie of *ex-vivo* cultured salivary glands co-expressing UAS-LifeAct-Ruby (green) and UAS-Whamy-eGFP (magenta) under the ptc-Gal4 driver. Scale bar 5µm.

Journal of Cell Science • Supplementary information

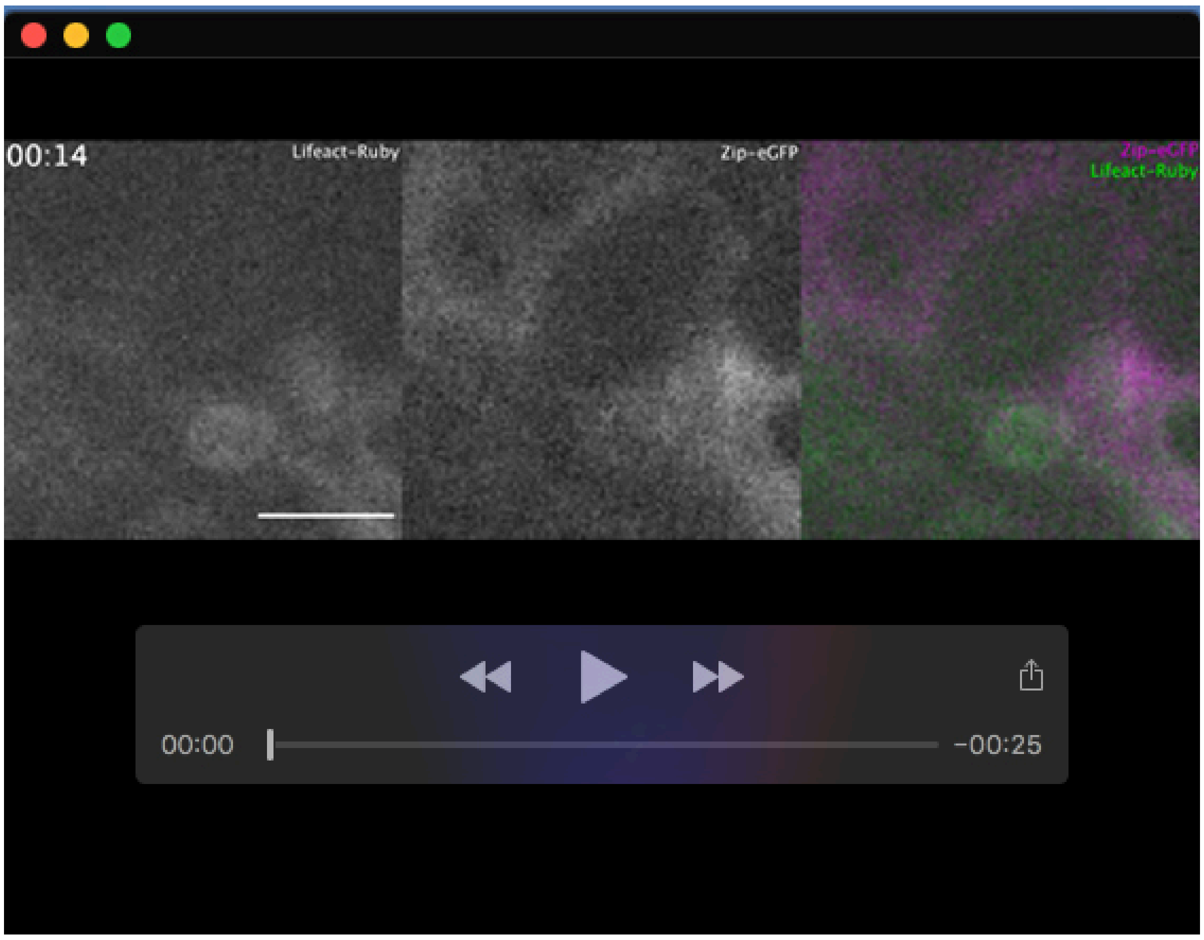

Movie 7. Representative time-lapse movie of *ex-vivo* cultured salivary glands co-expressing UAS-LifeAct-Ruby (green) under the ptc-Gal4 driver and zip-GFP (magenta). Scale bar 5μm.

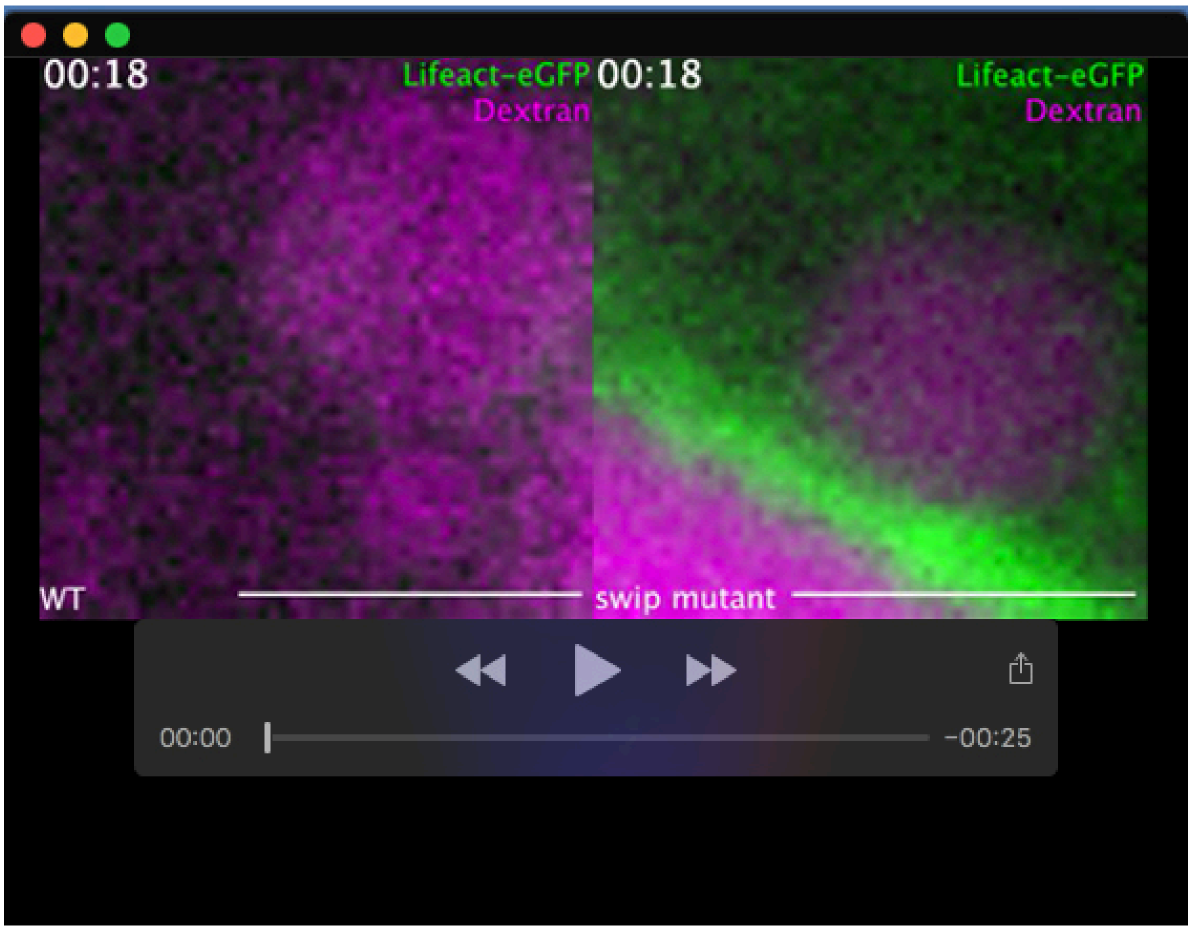

Movie 8. Time-lapse movie of *ex vivo* cultured wildtype (left) and *swip-1* mutant (right) salivary glands infused with A568-dextran (magenta) and expressing LifeAct-eGFP (green) under the *srp*-Gal4 driver. Scale bar 5μm.

Journal of Cell Science • Supplementary information

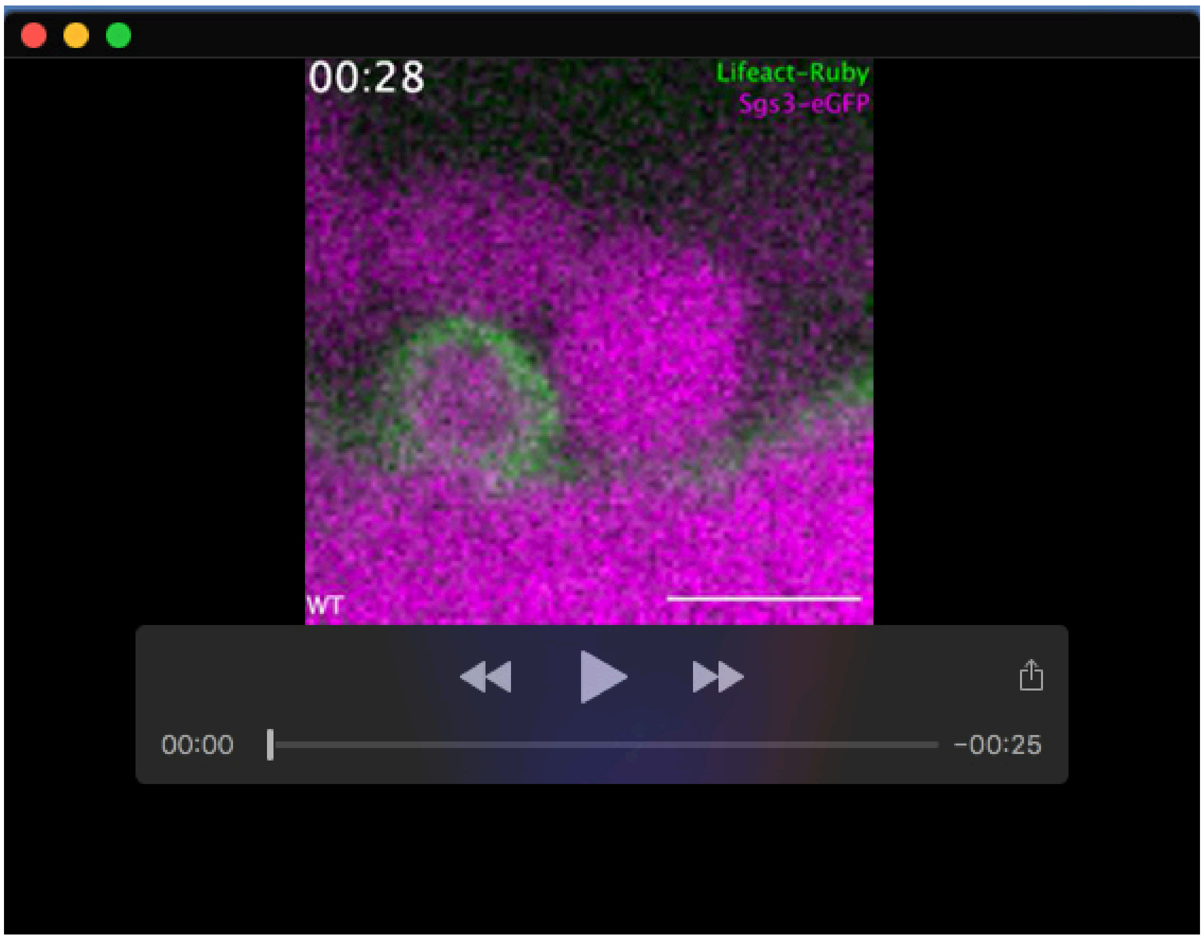

Movie 9. Representative time-lapse movie of *ex-vivo* cultured salivary glands co-expressing UAS-LifeAct-Ruby (green) under the ptc-Gal4 driver and Sgs3-GFP (magenta) in a wildtype genetic background. Scale bar 5µm

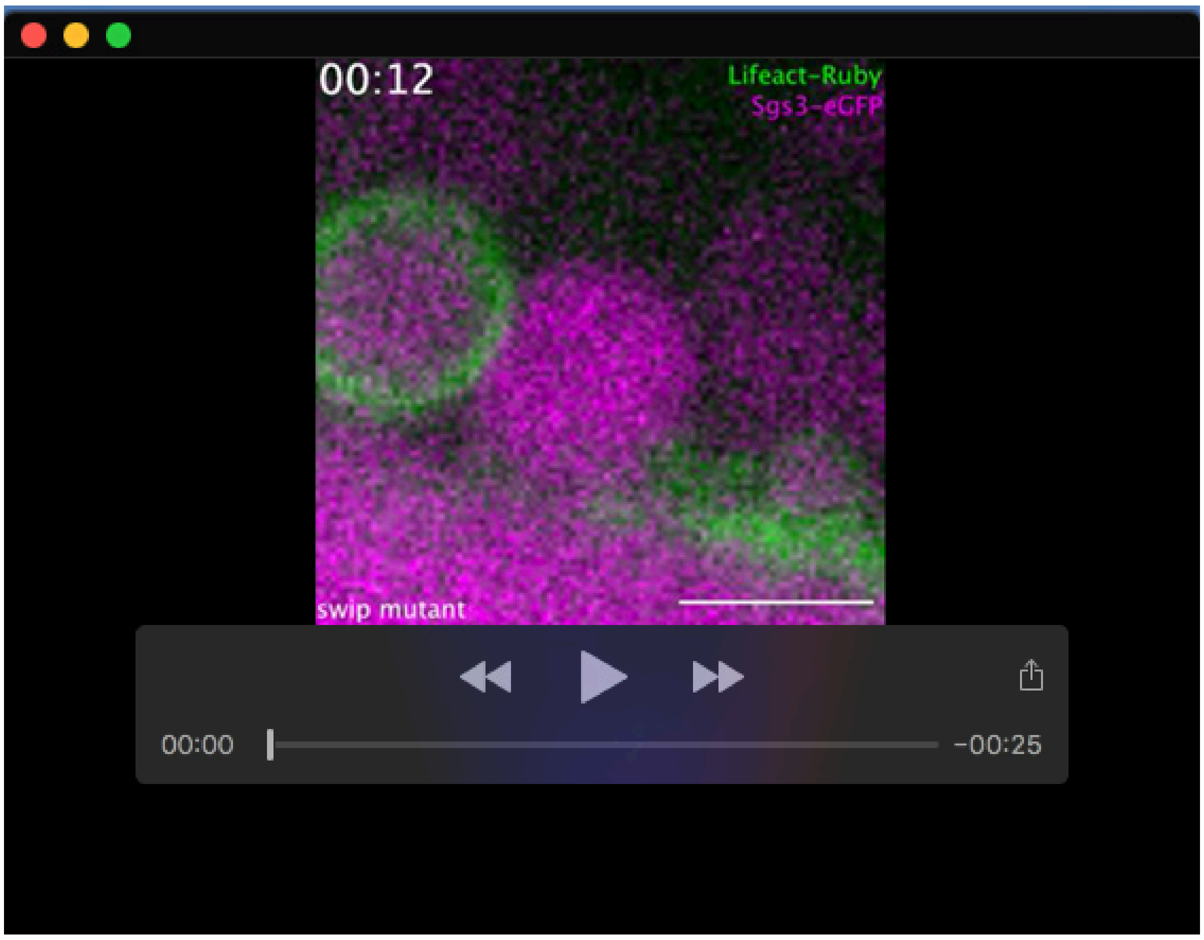

Movie 10. Representative time-lapse movie of ***ex-vivo* cultured salivary glands co-expressing UAS-LifeAct-Ruby (green) under the ptc-Gal4 driver and Sgs3-GFP (magenta) in a *swip-1* mutant genetic background.** Scale bar 5µm.

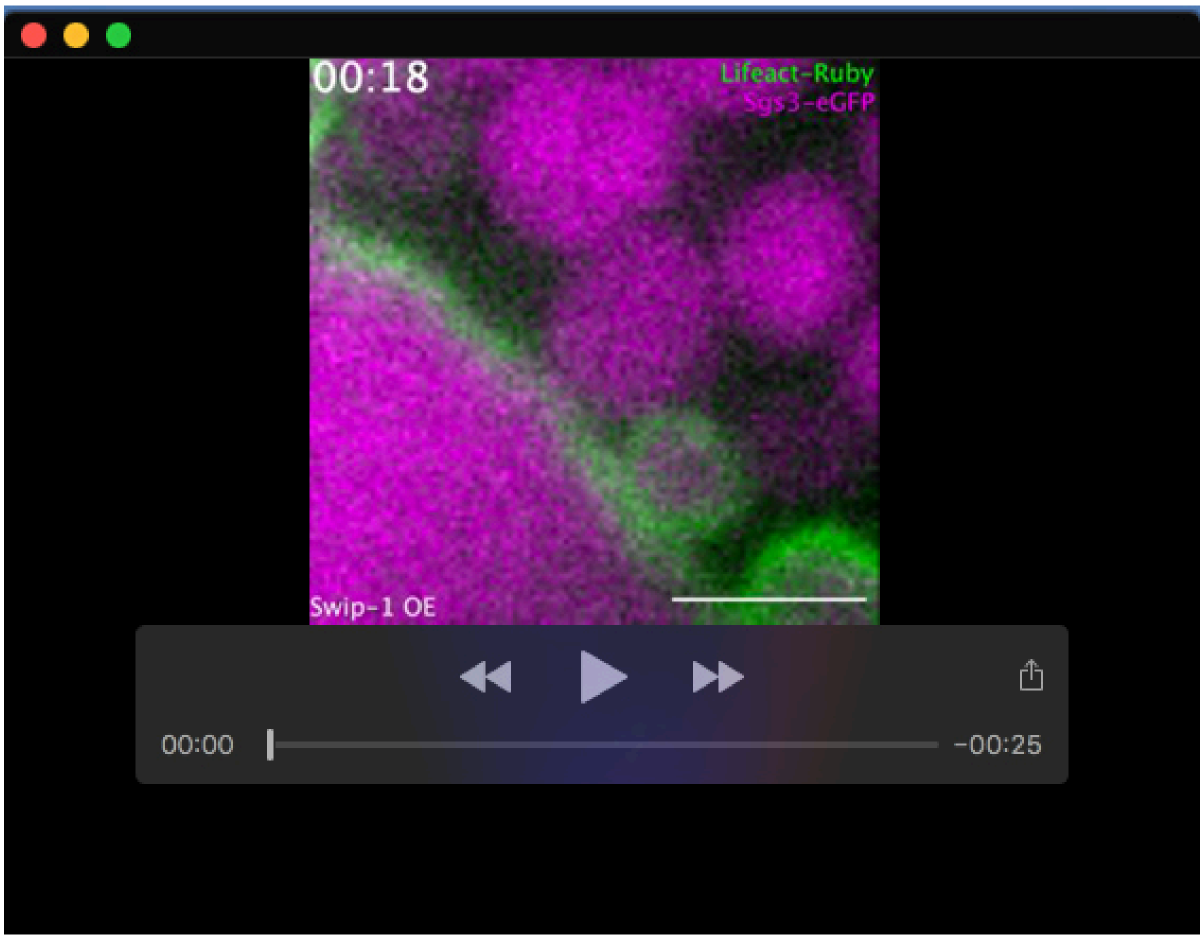

Movie 11. Representative time-lapse movie of *ex-vivo* cultured salivary glands co-expressing UAS-LifeAct-Ruby (green) and overexpressing UAS-Swip-1 under the ptc-Gal4 driver and Sgs3-GFP (magenta). Scale bar 5µm

Journal of Cell Science • Supplementary information
