# Supplementary material for: Swip-1 promotes exocytosis of glue granules in the exocrine Drosophila salivary gland
Source: J Cell Sci. 2023 Mar 6;136(6):jcs260366. doi: 10.1242/jcs.260366 (PMC10038153; doi:10.1242/jcs.260366)
Supplement: Supplementary file 1 [file joces-136-260366_review_history.pdf]

Swip-1 promotes exocytosis of glue granules in the exocrine *Drosophila* salivary gland

Franziska Lehne and Sven Bogdan
DOI: 10.1242/jcs.260366

Editor: Michael Way

Review timeline

Original submission:	22 June 2022
Editorial decision:	1 August 2022
First revision received:	28 December 2022
Editorial decision:	24 January 2023
Second revision received:	26 January 2023
Accepted:	27 January 2023

Original submission

First decision letter

MS ID#: JOCES/2022/260366

MS TITLE: EFhD2/Swip-1 promotes exocytosis of glue granules in the exocrine Drosophila salivary gland

AUTHORS: Sven Bogdan and Franziska Lehne
ARTICLE TYPE: Research Article

We have now reached a decision on the above manuscript.

To see the reviewers' reports and a copy of this decision letter, please go to: https://submit-jcs.biologists.organd click on the 'Manuscripts with Decisions' queue in the Author Area. (Corresponding author only has access to reviews.)

As you will see, the reviewers raise a large number of substantial criticisms and concerns. Given their comments, you will need to provide additional experiments to address their concerns including using SGS3-GFP a native cargo under the control of its endogenous promotor (this point was raised by both reviewers). If you think that you can deal satisfactorily with the criticisms on revision, I would be pleased to see a revised manuscript. We would then return it to the reviewers.

Please ensure that you clearly highlight all changes made in the revised manuscript. Please avoid using 'Tracked changes' in Word files as these are lost in PDF conversion.

I should be grateful if you would also provide a point-by-point response detailing how you have dealt with the points raised by the reviewers in the 'Response to Reviewers' box. Please attend to all of the reviewers' comments. If you do not agree with any of their criticisms or suggestions please explain clearly why this is so.

Reviewer 1

Advance summary and potential significance to field

© 2023. Published by The Company of Biologists under the terms of the Creative Commons Attribution License (https://creativecommons.org/licenses/by/4.0/).

This manuscript explores the role of actin cross-linker EFhD2/Swip-1 in regulated secretion. Lehne and Bogdan utilize the Drosophila larval salivary gland, and associated genetic and transgenic tools, to characterize the contribution and importance of Swip-1 to secretory granule secretion. The authors present data demonstrating the temporal and spatial recruitment of Swip-1 to secreting vesicles, as it relates to previously published and established components of the actin cytoskeleton, and the consequence of Swip-1 deletion to salivary gland secretion.

The importance of the actin cytoskeleton and its components to regulated secretion is immense and the authors utilize a unique experimental system to study how actin cross linking proteins could mechanistically contribute to this biologically relevant process.

Comments for the author

Although this manuscript offers potentially interesting insight into the role of actin cross linking proteins in granule secretion, there are serious issues with and numerous inconsistencies in the data presented in this manuscript that should be addressed before it can be considered for publication.

Major concerns:

1. The authors utilize SerpCBP-eGFP for their main assay to determine the importance of Swip-1 to salivary gland secretion and to define the regions of Swip-1 that could effectively rescue the phenotype (figure 3A). In this system there is no reason to over express an exogenous reporter to assay secretion. The authors should utilize SGS3-GFP, the native cargo that is under the control of its endogenous promotor, for this assay. Additionally, the use of a stereomicroscope to detect cargo in the lumen of salivary glands as a gauge of secretion is not sufficient. With widefield epifluorescence there is a threshold of cargo that needs to accumulate in the lumen before one can visualize its expansion and filling with cargo. The authors should score secretion with point scanning or spinning disk confocal where one can clearly see an empty lumen or individual secreting granules.

2. In the Swip-1 deletion line the authors present data showing that secretion of SGS3-GFP granules is delayed compared to WT, 380s +/- 177.6s vs 152.9s +/-56.5s (figure 3K). This result is used to support their main hypothesis. However, when the authors look at the kinetics of granule secretion and Swip-1 recruitment using their Swip-1-eGFP line it takes ~340s from pore formation to complete expulsion of the secretory granule (figure 2A). This is a major inconsistency. If the Swip-1-eGFP line behaves like the deletion line, in terms of rate of granule secretion, then the results from experiments utilizing one of those two lines are compromised. The authors need to address this issue.

3. The authors conclude that Lifeact and Swip-1 are recruited to secreting granules at the same time. The graph in figure 2C says otherwise. The Swip-1 profile increases before the Lifeact profile. What change in fluorescent intensity is used to define the time at which lifeact or Swip-1 is first seen on the granule? This question is relevant for all assays quantifying recruitment of factors to secreting granules.

4. The authors present a graph quantifying the time of recruitment of Lifeact vs Zip/WHAMY/Swip-1 to secreting granules (figure 2D). There are no representative images for Lifeact/Zip or Lifeact/WHAMY. This data is relevant and important. Representative time series should minimally be included in supplemental materials.

5. The authors suggest that loss of Swip-1 results in granules that fuse with the apical membrane but fail to collapse or collapse only after a significant delay. The authors do not present any images to support this conclusion. The graphs alone (figures 3J and 3K) are not sufficient to support the authors' statements. A time series showing SGS3-GFP/lifeact in Swip-1 depleted glands is essential for the authors to make this conclusion. The authors then compare their phenotype to what has been reported following depletion of Arp2/3-branched actin nucleators. The comparison of the Swip-1 phenotype to that of Arp2/3 and WASP-family protein depletion is incorrect, at least until the authors provide a representative time series to demonstrate the Swip-1 phenotype.

© 2023. Published by The Company of Biologists under the terms of the Creative Commons Attribution License (https://creativecommons.org/licenses/by/4.0/).

6. There is a large amount of variability in quantification of temporal events during granule secretion presented by the authors. This variability in replicates complicates analysis of the data and conclusions that can be made from the data. It is possible that motion artifact during imaging, which can be seen in the supplemental movies, might be affecting subsequent analysis. This is typically compounded when signal-to-noise is low. The authors should try to address this variability.

Concerns

A. The authors use western blots to characterize the specificity of their Swip-1 antibody. It would be nice to see antibody staining of Swip-1 depleted salivary glands. This would strengthen the authors' conclusions regarding Swip-1 localization before the onset of secretion.

B. The authors use their dextran experiments to draw conclusions about vesicle fusion. This isn't accurate. The dextran experiments identify the formation of the fusion pore, which occurs after fusion. The authors would need to utilize something similar to a PIP2 reporter (PLCdPH-GFP) to draw conclusions about granule fusion with the apical membrane.

C. The authors often describe non-secreting cells as non-secretory cells. This is confusing since the larval salivary gland is made of secretory cells, as shown by the authors in figure 1A.

Minor

I. A brief statement as to why srp-Gal4 was used would be informative.

II. Swip-1-eGFP is used in figure 2A and eGFP-Swip-1 is used in figure 2B. Is there a reason why the respective N- or C-terminally tagged protein isn't used for both experiments?

III. The authors describe the number of secretory events and salivary glands used for each experiment. A description of the number of independent crosses used would also be helpful.

IV. There are experiments that are described in the materials and methods that didn't seem to make it into the manuscript in figure form.

V. Supplemental movies are each formatted differently (scale bars, time stamp location, labeling). Consistent formatting would help in the consumption of the data.

VI. Supplemental movie M3 seems to have different magnifications for WT and Swip-1 depleted glands. This complicates the interpretation.

<u>Reviewer 2</u>

Advance summary and potential significance to field

In this manuscript, Lehne & Bogdan set out to characterize the role of the actin regulator Swip-1 in regulated exocytosis of glue proteins in the Drosophila larval salivary glands. The authors show that Swip-1 exhibits a dynamic localization to secreting vesicles similar to that of the F-actin reporter Lifeact. Swip-1 does not appear to be required for vesicle fusion or fusion pore formation, but instead, the authors suggest that it is required for expulsion of secretory cargo and collapse of secretory vesicles. Although the protein localization data seems generally sound, other major conclusions of the manuscript (primarily the role of Swip-1 during exocytosis) are not adequately supported by the presented data.

Comments for the author

Overall, the manuscript is lacking in depth of analysis and does not contain the level of experimental detail and rigor to adequately support the stated conclusions.

© 2023. Published by The Company of Biologists under the terms of the Creative Commons Attribution License (https://creativecommons.org/licenses/by/4.0/).

Major concerns:

1) The data in Fig. 3A-B do not adequately demonstrate that Swip-1 is required for exocytosis. Serp-CBP-eGFP is not known to be an endogenous secretory cargo in the larval salivary glands; thus, it is unclear why this secretory cargo was chosen for analysis when the endogenously regulated Sgs3-GFP reagent is publicly available (and was used in Fig. 3I-J). At minimum, authors should perform the ex vivo secretion assay using Sgs3-GFP. Importantly, however, there are significant concerns with reliance on the ex vivo assay without consideration for the systemic phenotypes of Swip-1 mutant animals. No information is included in this manuscript or in Lehne et al. 2022 about the developmental phenotypes of Swip-1 mutant animals. Are they lethal? Do they exhibit any developmental delays? Developmental delay phenotypes could account for the delayed onset of secretion shown in Fig. 3A-B, since the salivary glands must reach the correct developmental stage to acquire “competence” to respond to 20E, even in ex vivo culture. Therefore, analysis of secretion in Swip-1 mutant glands should be accompanied by analysis of secretion upon salivary gland-specific RNAi knockdown of Swip-1 (using the fkh-GAL4 or Sgs3-GAL4 driver). Furthermore, the authors could use an in vivo approach by isolating salivary glands from control and Swip-1 mutant animals at the onset of metamorphosis, when endogenous exocytosis of Sgs3-GFP is complete. Retention of Sgs3-GFP secretory vesicles in Swip-1 mutant animals at this timepoint would better support a requirement for this protein in exocytosis.

2) The authors state that Swip-1 is not required for vesicle fusion, and present the data in Fig. 3E to support this statement. The authors also state that Swip-1 appears to be required for vesicle contraction/collapse and release of secretory cargo. However, work by others (Rousso et al. 2016; Tran et al. 2015) shows that when vesicle contraction is defective (via disruption of myosin II or Arp2/3), very large vesicles form and are not secreted. These large vesicles likely result from expansion and hydration of the hygroscopic mucin cargo. The authors should report if this enlarged vesicle phenotype is observed in Swip-1 mutant glands. Without this phenotype, it is difficult to reconcile the authors’ conclusion that Swip-1 acts during vesicle collapse and cargo release with the previous results published in the literature.

3) The authors suggest in Fig. 4B that secreting vesicles in Swip-1 mutant glands have higher levels of active myosin II. This statement should be supported by additional data, including quantification of the p-MRLC staining and timelapse imaging of myosin-II in control and Swip-1 mutant salivary gland cells (using publicly available zip-GFP and/or sqh-GFP/sqh-mCherry). Additionally, Rousso et al. 2016 shows that myosin II is still recruited to secreting vesicles upon Arp2/3 knockdown, but the organization of myosin is disrupted. The data shown in this manuscript again conflict with previously published work, and the authors need to explain how their findings (an increased level of myosin-II on secreting vesicles in Swip-1 mutant glands) result in secretion defects.

4) Additional analysis of the sequence of Swip-1 localization to secreting vesicles relative to other known regulators should be included. The authors should perform their own analysis of the timing of Arp3 localization to secreting granules instead of referencing previously published values (lines 172-174), especially since a UAS-Arp3-GFP reagent is publicly available. The sequence of recruitment of Swip-1 to secreting vesicles relative to other known markers is a major point of the manuscript, and the authors rely on this sequence of events to determine how Swip-1 regulates secretion. Thus, a rigorous examination of this sequence is necessary to support the stated conclusions of the manuscript. The authors should also analyze the timing of Swip-1 localization with at least one other marker besides Lifeact. A sqh-mCherry construct is publicly available and would allow direct analysis of the relative timing of Swip-1 and myosin II to secreting granules.

5) The calcium-related data described in the Discussion section (lines 267-274) should be included in the manuscript. This data would help to provide additional experimental depth to the manuscript, and also lend more credence to the argument that the dimerization domain, and not the calcium-binding domain, of Swip-1 is required for its role in secretion.

Additional minor comments:

1) There is a general lack of introductory info about the Swip-1 protein. The authors should include information about the structure and known functions of this protein in the Introduction.

© 2023. Published by The Company of Biologists under the terms of the Creative Commons Attribution License (https://creativecommons.org/licenses/by/4.0/).

2) Experimental replicate details should be included in the legends for Fig. 1, Fig. 2A-B, and Fig. 4A-B.

3) The statement in lines 255-257 of the Discussion section is incorrect. Synthesis (not regulated exocytosis) of mucin-like glue proteins is triggered by a small pulse of 20E at the mid-third instar transition. Exocytosis of mucins is triggered by the late larval pulse of 20E that also triggers the onset of metamorphosis.

4) Removal of the calcium data from the Discussion section (see point 5, above) will leave room for the authors to further elaborate on the potential role of Swip-1 in actin crosslinking and/or actin filament sliding and to better describe the potential roles of the Swip-1 protein domains in these processes.

First revision

<u>Author response to reviewers' comments</u>

We would like to thank all reviewers for careful and thorough reading of our manuscript as well as their constructive suggestions and comments. To the best of our possibilities, we have revised our manuscript accordingly, and are optimistic that the made changes strengthen our conclusions and improve the quality of the manuscript.

Let me briefly highlight the **most important points** that we have addressed before providing a detailed point-to-point- response to the referees' comments.

1. As suggested by both reviewers, we have now established numerous new recombined fly stocks expressing the SGS3-GFP reporter as a native cargo under the control of its endogenous promotor. As shown in Figure 3A, B, using this new reporter assay we could confirm our major finding that EFhD2/Swip-1 promotes secretory vesicle compression and expulsion of cargo during regulated exocytosis. *swip-1* null mutant salivary glands showed a striking delayed secretion. Compared to wild type, *swip-1* null mutant salivary glands showed a striking delayed secretion with most glands secreting after six hours post induction (Figure 3B). Again, impaired secretion of *swip-1* mutant salivary glands was fully rescued by re-expression of a full-length Swip-1 transgene (Figure 3B).

2. As suggested by one reviewer, we now carefully quantified possible developmental delay in larval to pupal development. We found that there is no significant difference between wild type and *swip*Δ1 animals at any time point of larval-to-pupal transition (metamorphosis). Thus, developmental delay does not account for delayed secretion of *swip-1* null mutant salivary glands.

3. Our new rescue experiments revealed that the function of EFhD2/Swip-1 in regulating secretory cargo expulsion neither require calcium-binding nor dimerization of EFhD2/Swip-1. We now provided further evidence that independent of its calcium- dependent cross-linking activity EFhD2/Swip-1 might play an additional important role in regulating Rho-GTPase signaling. Supporting this notion, we found that loss of EFhD2/Swip-1 results in a significant premature recruitment of Rho to fused secreting vesicles that explains a dramatic increase of active myosin by 4.5-fold as now quantified in *swip*Δ1 mutant salivary glands in the revised manuscript.

4. Finally, we provided new evidence that loss of EFhD2/Swip-1 function affects actomyosin-mediated vesicular membrane crumpling, a recently described mechanism which helps to squeeze the content out of the vesicle while retaining and sequestering the vesicle membrane. Both, *swip-1* mutant and Swip-1 overexpressing salivary glands showed reduced membrane crumpling although less severe compared to zip-RNAi (myosin II). These new data are included in new figure 4L.

REVIEWERS' COMMENTS

Reviewer 1 Advance Summary and Potential Significance to Field: This manuscript explores the role of actin cross-linker EFhD2/Swip-1 in regulated secretion. Lehne and Bogdan utilize the Drosophila larval salivary gland, and associated genetic and transgenic tools, to characterize the contribution and importance of Swip-1 to secretory granule secretion. The authors

© 2023. Published by The Company of Biologists under the terms of the Creative Commons Attribution License (https://creativecommons.org/licenses/by/4.0/).

present data demonstrating the temporal and spatial recruitment of Swip-1 to secreting vesicles, as it relates to previously published and established components of the actin cytoskeleton, and the consequence of Swip-1 deletion to salivary gland secretion.

The importance of the actin cytoskeleton and its components to regulated secretion is immense and the authors utilize a unique experimental system to study how actin cross linking proteins could mechanistically contribute to this biologically relevant process.

We agree and thank the reviewer for pointing this out.

Reviewer 1 Comments for the Author: Although this manuscript offers potentially interesting insight into the role of actin cross linking proteins in granule secretion, there are serious issues with and numerous inconsistencies in the data presented in this manuscript that should be addressed before it can be considered for publication.

Major concerns:

1. The authors utilize SerpCBP-eGFP for their main assay to determine the importance of Swip-1 to salivary gland secretion and to define the regions of Swip-1 that could effectively rescue the phenotype (figure 3A). In this system there is no reason to over express an exogenous reporter to assay secretion. The authors should utilize SGS3-GFP, the native cargo that is under the control of its endogenous promotor, for this assay.

We agree and we have now established numerous new recombined fly stocks expressing the SGS3-GFP reporter as a native cargo under the control of its endogenous promotor. As shown in new Figure 3A, B, using this new reporter assay we could confirm our major finding that EFhD2/Swip-1 promotes secretory vesicle compression and expulsion of cargo during regulated exocytosis. *swip-1* null mutant salivary glands showed a striking delayed secretion, and even after eight hours post treatment only half of the mutant glands became secretory. Again, impaired secretion of *swip-1* mutant salivary glands was fully rescued by re-expression of a full-length Swip-1 transgene (new Figure 3B).

Additionally, the use of a stereomicroscope to detect cargo in the lumen of salivary glands as a gauge of secretion is not sufficient. With widefield epifluorescence there is a threshold of cargo that needs to accumulate in the lumen before one can visualize its expansion and filling with cargo. The authors should score secretion with point scanning or spinning disk confocal where one can clearly see an empty lumen or individual secreting granules.

We agree and we have now quantified all phenotypes using the spinning disk confocal microscope (see also new figure 3A).

2. In the Swip-1 deletion line the authors present data showing that secretion of SGS3-GFP granules is delayed compared to WT, 380s +/- 177.6s vs 152.9s +/-56.5s (figure 3K). This result is used to support their main hypothesis. However, when the authors look at the kinetics of granule secretion and Swip-1 recruitment using their Swip-1-eGFP line it takes ~340s from pore formation to complete expulsion of the secretory granule (figure 2A). This is a major inconsistency. If the Swip-1-eGFP line behaves like the deletion line, in terms of rate of granule secretion, then the results from experiments utilizing one of those two lines are compromised. The authors need to address this issue.

We thank the reviewer for pointing this out and followed up this suggestion. Interestingly, we indeed found that the overexpression of EFhD2/Swip-1 resulted in a similar prolonged cargo release suggesting that increased EFhD2/Swip-1 protein level interferes with its function. We now included the overexpression data and quantifications in new Figure 4D, E, F and supplementary movie M11.

3. The authors conclude that Lifeact and Swip-1 are recruited to secreting granules at the same time. The graph in figure 2C says otherwise. The Swip-1 profile increases before the Lifeact profile. What change in fluorescent intensity is used to define the time at which lifeact or Swip-1 is first seen on the granule? This question is relevant for all assays quantifying recruitment of factors to secreting granules.

© 2023. Published by The Company of Biologists under the terms of the Creative Commons Attribution License (https://creativecommons.org/licenses/by/4.0/).

We apologize for unclear description and included a more detailed description in the material & methods section. Measurement of percent fluorescent intensity of two proteins of interest (e.g. LifeAct/Swip-1) was performed as previously described (Tran et al., 2015). In short, an oval region of interest was drawn around single fusing vesicles in a single z-plane. The "plot z-axis profile" function in ImageJ was used to obtain fluorescence intensity values for each channel. Subsequent calculations of % fluorescence intensity and time difference of detection were carried out in Microsoft Excel according to Tran and colleagues. Start of protein recruitment to the vesicle membrane was defined as fluorescence intensity increase of ≤1% compared to the previous frame. Accordingly, measurement of time of cargo expulsion was determined by calculating the time difference between detection of Lifeact and minimal Sgs3 fluorescence in the region of interest. For data plotting, Sgs3 fluorescence at onset of actin coat formation was set to 100% and minimal detection of fluorescence at end of cargo expulsion (i.e. background fluorescence) set to 0%.

In addition, we further quantified Lifeact and Swip-1 recruitment in relation to Arp3- GFP as a reference. As shown in the new figure 2D, Lifeact and Swip-1 are both detected at the vesicle membrane at the same time in relation to Arp3 detection. LifeAct: -5.48s ± 10.87s, n= 23 vesicles from 7 SG and Swip-1: -5.68s ± 11.04s, n= 24 vesicles from 6 SGs.

4. The authors present a graph quantifying the time of recruitment of Lifeact vs Zip/WHAMY/Swip-1 to secreting granules (figure 2D). There are no representative images for Lifeact/Zip or Lifeact/WHAMY. This data is relevant and important. Representative time series should minimally be included in supplemental materials.

We apologize for unclear description and further provided these important data and information in figure 2C (quantification of Swip1, Rho, Arp3, Whamy, Dia and Zip recruitment in relation to LifeAct). We now also included representative time series of all proteins as new supplementary movies M3, M4, M5, M6 and M7.

5. The authors suggest that loss of Swip-1 results in granules that fuse with the apical membrane but fail to collapse or collapse only after a significant delay. The authors do not present any images to support this conclusion. The graphs alone (figures 3J and 3K) are not sufficient to support the authors' statements. A time series showing SGS3-GFP/lifeact in Swip-1 depleted glands is essential for the authors to make this conclusion. The authors then compare their phenotype to what has been reported following depletion of Arp2/3-branched actin nucleators. The comparison of the Swip-1 phenotype to that of Arp2/3 and WASP-family protein depletion is incorrect, at least until the authors provide a representative time series to demonstrate the Swip-1 phenotype.

We now provided the missing representative graphs of Lifeact recruitment and Sgs3 expulsion in wild type, *swip-1* mutant and Swip-1 overexpressing salivary glands in the new figure 4E. Quantification of expulsion duration defined as time from actin coat formation to minimal Sgs3-GFP detection is now shown in figure 4F. Cargo expulsion is significantly delayed in both *swip-1* mutants and Swip-1 overexpression.

6. There is a large amount of variability in quantification of temporal events during granule secretion presented by the authors. This variability in replicates complicates analysis of the data and conclusions that can be made from the data. It is possible that motion artifact during imaging, which can be seen in the supplemental movies, might be affecting subsequent analysis. This is typically compounded when signal-to-noise is low. The authors should try to address this variability.

We further increased the replicates and additionally included quantification of Rho, Arp3 and Dia and Zip recruitment in relation to LifeAct; see new supplementary movies M3, M4, M5, M6 and M7).

Concerns

A. The authors use western blots to characterize the specificity of their Swip-1 antibody. It would be nice to see antibody staining of Swip-1 depleted salivary glands. This would strengthen the authors' conclusions regarding Swip-1 localization before the onset of secretion.

© 2023. Published by The Company of Biologists under the terms of the Creative Commons Attribution License (https://creativecommons.org/licenses/by/4.0/).

We now included the missing immunostainings in new figure 1D. A complete loss of immunostaining in mutant salivary glands confirmed the high specificity of the anti- Swip-1 antibody (Figure 1D, D'').

B. The authors use their dextran experiments to draw conclusions about vesicle fusion. This isn't accurate. The dextran experiments identify the formation of the fusion pore, which occurs after fusion. The authors would need to utilize something similar to a PIP2 reporter (PLCdPH-GFP) to draw conclusions about granule fusion with the apical membrane.

The reviewer is correct, our dextran experiments identify the formation of the fusion pore, which occurs after fusion. Since mutant SGs showed no defective vesicle fusion pore formation, we would not expect any significant defects in vesicle fusion. Therefore, we changed the text accordingly.

C. The authors often describe non-secreting cells as non-secretory cells. This is confusing since the larval salivary gland is made of secretory cells, as shown by the authors in figure 1A.

The reviewer is correct and we changed the text accordingly.

Minor
I. A brief statement as to why srp-Gal4 was used would be informative.

Srp-Gal4 already drives robust expression from the early larva (L1, L2) to late stages as previously described in Gyoergy et al., 2018.

Gyoergy, A., M. Roblek, A. Ratheesh, K. Valoskova, V. Belyaeva, S. Wachner, Y. Matsubayashi, B.J. Sanchez-Sanchez, B. Stramer, and D.E. Siekhaus. 2018. Tools Allowing Independent Visualization and Genetic Manipulation of Drosophila melanogaster Macrophages and Surrounding Tissues. G3 (Bethesda). 8:845-857.

II. Swip-1-eGFP is used in figure 2A and eGFP-Swip-1 is used in figure 2B. Is there a reason why the respective N- or C-terminally tagged protein isn't used for both experiments?

The reviewer is correct, there is no clear reason, both N- or C-terminally tagged fusion worked well. Therefore, we now presented only the C-terminal tagged version (see new figure 2B.

III. The authors describe the number of secretory events and salivary glands used for each experiment. A description of the number of independent crosses used would also be helpful.

We agree and included this information in the figure legends.

IV. There are experiments that are described in the materials and methods that didn't seem to make it into the manuscript in figure form.

We apologize for potentially confusing information and removed it.

V. Supplemental movies are each formatted differently (scale bars, time stamp location, labeling). Consistent formatting would help in the consumption of the data.

We now provided new supplemental movies with consistent format.

VI. Supplemental movie M3 seems to have different magnifications for WT and Swip-1 depleted glands. This complicates the interpretation.

We changed this in the new figure 4E and in the supplementary movie (now M8) accordingly.

Reviewer 2 Advance Summary and Potential Significance to Field:

In this manuscript, Lehne & Bogdan set out to characterize the role of the actin regulator Swip-1 in

© 2023. Published by The Company of Biologists under the terms of the Creative Commons Attribution License (https://creativecommons.org/licenses/by/4.0/).

regulated exocytosis of glue proteins in the Drosophila larval salivary glands. The authors show that Swip-1 exhibits a dynamic localization to secreting vesicles similar to that of the F-actin reporter Lifeact. Swip-1 does not appear to be required for vesicle fusion or fusion pore formation, but instead, the authors suggest that it is required for expulsion of secretory cargo and collapse of secretory vesicles. Although the protein localization data seems generally sound, other major conclusions of the manuscript (primarily the role of Swip-1 during exocytosis) are not adequately supported by the presented data.

Reviewer 2 Comments for the Author:
Overall, the manuscript is lacking in depth of analysis and does not contain the level of experimental detail and rigor to adequately support the stated conclusions.

Major concerns:

1) The data in Fig. 3A-B do not adequately demonstrate that Swip-1 is required for exocytosis. Serp-CBP-eGFP is not known to be an endogenous secretory cargo in the larval salivary glands; thus, it is unclear why this secretory cargo was chosen for analysis when the endogenously regulated Sgs3-GFP reagent is publicly available (and was used in Fig. 3I-J). At minimum, authors should perform the ex vivo secretion assay using Sgs3-GFP.

We agree and we have now established numerous new recombined fly stocks expressing the SGS3-GFP reporter as a native cargo under the control of its endogenous promotor. As shown in new Figure 3A, B, using this new reporter assay we could confirm our major finding that EFhD2/Swip-1 promotes secretory vesicle compression and expulsion of cargo during regulated exocytosis. Compared to wild type, swip-1 null mutant salivary glands showed a striking delayed secretion with most glands secreting after six hours post induction (Figure 3B). Again, impaired secretion of *swip-1* mutant salivary glands was fully rescued by re-expression of a full-length Swip-1 transgene (new Figure 3B).

Importantly, however, there are significant concerns with reliance on the ex vivo assay without consideration for the systemic phenotypes of Swip-1 mutant animals. No information is included in this manuscript or in Lehne et al. 2022 about the developmental phenotypes of Swip-1 mutant animals. Are they lethal? Do they exhibit any developmental delays?

We apologize for the unclear description and we now provide more information about *swip-1* mutant. In detail, loss of EFhD2/Swip-1 function showed prominent defects in the innate immune system and epithelial wound closure (Lehne et al., 2022), but mutant flies are fully viable and fertile. In addition, we now carefully quantified possible developmental delays in larval to pupal development. However, we found there are no significant differences between wild type and *swip*Δ1 animals at any time point of larval-to-pupal transition. Thus, developmental delay does not account for delayed secretion in *swip-1* null mutant salivary glands. We included these data in supplemental figure 1).

Developmental delay phenotypes could account for the delayed onset of secretion shown in Fig. 3A-B, since the salivary glands must reach the correct developmental stage to acquire “competence” to respond to 20E, even in ex vivo culture. Therefore, analysis of secretion in Swip-1 mutant glands should be accompanied by analysis of secretion upon salivary gland-specific RNAi knockdown of Swip-1 (using the fkh-GAL4 or Sgs3-GAL4 driver).

As mentioned above, developmental delay does not account for delayed secretion *swip-1* null mutant salivary glands. As suggested by the reviewer we tried to suppress EFhD2/Swip-1 function by expressing a RNAi construct under the control of the *sgs*3- Gal4 driver. Unfortunately, Western blot analysis revealed an incomplete knockdown which did not allow clear conclusions.

Furthermore, the authors could use an in vivo approach by isolating salivary glands from control and Swip-1 mutant animals at the onset of metamorphosis, when endogenous exocytosis of Sgs3-GFP is complete. Retention of Sgs3-GFP secretory vesicles in Swip-1 mutant animals at this timepoint would better support a requirement for this protein in exocytosis.

© 2023. Published by The Company of Biologists under the terms of the Creative Commons Attribution License (https://creativecommons.org/licenses/by/4.0/).

We thank the reviewer for this suggestion. We followed up this idea. However, it turned out that the massive histolysis of larval tissue including SGs immediately starts after completing SG secretion. Thus, it is not possible to obtain reliable data at the onset of metamorphosis.

2) The authors state that Swip-1 is not required for vesicle fusion, and present the data in Fig. 3E to support this statement. The authors also state that Swip-1 appears to be required for vesicle contraction/collapse and release of secretory cargo. However, work by others (Rousso et al. 2016; Tran et al. 2015) shows that when vesicle contraction is defective (via disruption of myosin II or Arp2/3), very large vesicles form and are not secreted. These large vesicles likely result from expansion and hydration of the hygroscopic mucin cargo. The authors should report if this enlarged vesicle phenotype is observed in Swip-1 mutant glands. Without this phenotype, it is difficult to reconcile the authors' conclusion that Swip-1 acts during vesicle collapse and cargo release with the previous results published in the literature.

We thank the reviewer for this interesting question. We see that individual wild type secretory vesicles undergo a slight expansion directly after fusion with the apical PM, likely due to hydration-related expansion of mucins creating mechanical forces that will be counter-balanced by the branched actomyosin vesicle coat (Tran et al, 2015). We did not see very large vesicles formed in *swip-1* null mutant salivary glands as reported for *arp2/3* knockdown. As suggested by Tran and colleagues, the fused secretory vesicles continue to expand in size in the absence of branched actin, likely due to reduced counter-balancing actin coat. However, in *swip-1* mutants the situation is different. We provided further evidence that loss of EFhD2/Swip-1 results in a significant premature recruitment of Rho to fused secreting vesicles that explains the dramatic increase of active myosin by 4.5-fold observed in *swipΔ1* mutant salivary glands in the revised manuscript (see new figure 4H, I). Thus, increased level of active myosin might reduce myosin dynamics and further increase vesicle stiffness resulting in an inefficient vesicle compression and secretion in swipΔ1 mutant salivary glands. Inefficient compression also affects membrane crumpling, a recently described mechanism which helps to squeeze the content out of the vesicle while retaining and sequestering the vesicle membrane (Kamalesh et al., 2021). Both, swip-1 mutant and Swip-1 overexpressing salivary glands even showed reduced membrane crumpling although less severe compared to zip-RNAi (myosin II). These new data are included in new figure 4L.

3) The authors suggest in Fig. 4B that secreting vesicles in Swip-1 mutant glands have higher levels of active myosin II. This statement should be supported by additional data, including quantification of the p-MRLC staining and timelapse imaging of myosin- II in control and Swip-1 mutant salivary gland cells (using publicly available zip-GFP and/or sqh-GFP/sqh-mCherry).

As mentioned above, we quantified p-MRLC staining and found a dramatic increase of active myosin by 4.5-fold observed in swipΔ1 mutant salivary glands (see new figure 4H, I). We also performed time-lapse imaging of myosin-II using sqh-mCherry in wild type and *swip-1* mutant cells. In this experiment Sqh-mCherry appeared slightly but not significantly earlier on vesicles in swipΔ1 mutants compared to wildtype. However, we observed significant differences in Sqh-mCherry recruitment between in rescued *swip-1* mutant cells and cells overexpressing Swip-1. Consistent with our model, Sqh- mCherry localizes significantly earlier on vesicles in rescued *swip*Δ1 mutants compared to Swip-1 overexpression (included in supplemental figure 1).

Additionally, Rousso et al. 2016 shows that myosin II is still recruited to secreting vesicles upon Arp2/3 knockdown, but the organization of myosin is disrupted. The data shown in this manuscript again conflict with previously published work, and the authors need to explain how their findings (an increased level of myosin-II on secreting vesicles in Swip-1 mutant glands) result in secretion defects.

As described above, we propose that increased level of active myosin reduces myosin dynamics and further increases vesicle stiffness resulting in an inefficient vesicle compression and secretion in swipΔ1 mutants. Thus, we hypothesized that EFhD2/Swip-1 contributes to the recruitment of Rho-GTPase regulating actomyosin activity to drive proper vesicle membrane crumpling and expulsion of cargo.

4) Additional analysis of the sequence of Swip-1 localization to secreting vesicles relative to other known regulators should be included. The authors should perform their own analysis of the timing of

© 2023. Published by The Company of Biologists under the terms of the Creative Commons Attribution License (https://creativecommons.org/licenses/by/4.0/).

Arp3 localization to secreting granules instead of referencing previously published values (lines 172-174), especially since a UAS-Arp3- GFP reagent is publicly available. The sequence of recruitment of Swip-1 to secreting vesicles relative to other known markers is a major point of the manuscript, and the authors rely on this sequence of events to determine how Swip-1 regulates secretion. Thus, a rigorous examination of this sequence is necessary to support the stated conclusions of the manuscript. The authors should also analyze the timing of Swip-1 localization with at least one other marker besides Lifeact. A sqh-mCherry construct is publicly available and would allow direct analysis of the relative timing of Swip-1 and myosin II to secreting granules.

We agree with the reviewer and we further provided these important data in figure 2C (quantification of Swip1, Rho, Arp3, Whamy, Dia and Zip recruitment in relation to Lifeact). We now included representative time series of all proteins as new supplementary movies M3, M4, M5, M6 and M7. These data further support the two- step model, but also indicates a central role of the Rho-GTPase in the initial F-actin coat formation. First, Dia forms an initial linear actin filament coat. Second, Arp2/3 and its activators such as WASP, are subsequently recruited to promote the formation of branched actin coat structures. Together with myosin II this actin meshwork provides the mechanical forces needed to fold the membrane and thereby squeeze the content into the apical lumen (Rousso et al., 2016; Tran et al., 2015). Our new localization data suggests that Rho precedes Dia recruitment. Rho promotes Dia-mediated actin nucleation (Bogdan et al., 2013; Kuhn and Geyer, 2014; Spiering and Hodgson, 2011), but also promotes actomyosin contractility through activation of the Rho-dependent kinase Rok (Amano et al., 1996; Hodge and Ridley, 2016). We also further confirmed that EFhD2/Swip-1 is recruited simultaneously with F-actin to fused secretory vesicles.

5) The calcium-related data described in the Discussion section (lines 267-274) should be included in the manuscript. This data would help to provide additional experimental depth to the manuscript, and also lend more credence to the argument that the dimerization domain, and not the calcium-binding domain, of Swip-1 is required for its role in secretion.

We thank the reviewer for this suggestion. Given that calcium-binding is not crucial for EFhD2/Swip-1 function in SG secretion, we decided to remove the calcium-related data from the manuscript.

Additional minor comments:

1) There is a general lack of introductory info about the Swip-1 protein. The authors should include information about the structure and known functions of this protein in the Introduction.

We now provided more information about the structure and known functions of this protein in the manuscript.

2) Experimental replicate details should be included in the legends for Fig. 1, Fig. 2A- B, and Fig. 4A-B.

We agree and included this information in the figure legends.

3) The statement in lines 255-257 of the Discussion section is incorrect. Synthesis (not regulated exocytosis) of mucin-like glue proteins is triggered by a small pulse of 20E at the mid-third instar transition. Exocytosis of mucins is triggered by the late larval pulse of 20E that also triggers the onset of metamorphosis.

We agree and changed the text accordingly.

4) Removal of the calcium data from the Discussion section (see point 5, above) will leave room for the authors to further elaborate on the potential role of Swip-1 in actin crosslinking and/or actin filament sliding and to better describe the potential roles of the Swip-1 protein domains in these processes

As described above, we removed the calcium-related data from the manuscript.

© 2023. Published by The Company of Biologists under the terms of the Creative Commons Attribution License (https://creativecommons.org/licenses/by/4.0/).

Second decision letter

MS ID#: JOCES/2022/260366

MS TITLE: EFhD2/Swip-1 promotes exocytosis of glue granules in the exocrine Drosophila salivary gland

AUTHORS: Sven Bogdan and Franziska Lehne
ARTICLE TYPE: Research Article

We have now reached a decision on the above manuscript.

To see the reviewers' reports and a copy of this decision letter, please go to: https://submit-jcs.biologists.org and click on the 'Manuscripts with Decisions' queue in the Author Area. (Corresponding author only has access to reviews.)

As you will see, reviewer 2 is satisfied and recommends publication. In contrast, reviewer 1 raises a number of important points concerning your analysis. Having looked at your paper again, I am afraid I think these are important issues and will require amendments and additional analysis before I can accept your paper. If you think that you can deal satisfactorily with the criticisms on revision, I would be pleased to see a revised manuscript.

Please ensure that you clearly highlight all changes made in the revised manuscript. Please avoid using 'Tracked changes' in Word files as these are lost in PDF conversion.

I should be grateful if you would also provide a point-by-point response detailing how you have dealt with the points raised by the reviewers in the 'Response to Reviewers' box. Please attend to all of the reviewers' comments. If you do not agree with any of their criticisms or suggestions please explain clearly why this is so.

Reviewer 1

Advance summary and potential significance to field

The summary of the advance made and potential significance to the field are identical to the primary submission

Comments for the author

In the revised version of this manuscript Lehne and Bogdan provide several key experimental and textual changes to their primary submission. However, this revision still does not appropriately address the main concerns I have. The inconsistencies in the data and conclusions are still too numerous for publication.

Major Concerns:

1. The authors have now utilized SGS3-GFP to assay for the importance of Swip-1 to salivary gland secretion using spinning disk microscopy to visualize secretion. However, the data in figure 3B suggests that there is a subtle delay in secretion. There is 100% (within error) secretion in WT by 5 hrs and by 6 hrs in the swip-1 null. The authors state that "… and even after eight hours post treatment only half of the mutant glands become secretory" but figure 4B shows 100% secretion at the 8hr time point (full green bar, green secreting and black non-secreting) in the swip-1 null. Additionally, the authors state in the legends that "apoptotic and non-secreting (un-primed) SGs were discarded from analysis." If the point of the experiment is to see if glands secrete cargo, how can one discard non-secreting glands from the analysis?

2. The authors acknowledge and include new data showing that overexpression of Swip-1 results in prolonged cargo release that is similar to the Swip-1 null phenotype "suggesting that increased EFhD2/Swip-1 protein level interferes with its function." Because this is that case the results from

© 2023. Published by The Company of Biologists under the terms of the Creative Commons Attribution License (https://creativecommons.org/licenses/by/4.0/).

the Swip-1-eGFP experiments (figure 2) are compromised. Swip-1-eGFP is overexpressed to visualize when the protein is recruited to secreting cargo, related to lifeact and other markers. However, if Swip-1 overexpression prolongs the secretory event this experiment can't be compared to Zip/Lifeact, Whamy/Lifeact Dia/Lifeact, etc.

3. In figure 2C the authors conclude that Swip-1 is recruited to secreting granules before lifeact. However, in figure 2D the authors show that the time of recruitment of Swip-1 vs Arp3 is identical to that of Lifeact vs Arp3 and thus conclude both lifeact and Swip-1 are recruited simultaneously to the secreting vesicle. I am unsure how the authors come to their final conclusion here and why the data involving Arp3 supersedes that of the Swip-1/Lifeact (2C) experiment.
The time relationship between Lifeact and Arp3 is a fixed linear event. If Swip-1 is seen on granules before lifeact (2C) the same must hold true when both are individually compared to Arp3. The difference in result is likely due to variability in the analysis and large error seen in the individual replicates. See item 6 below.

4. The authors have added appropriate supplemental movies.

5. Removal of the phenotypic comparison between Swip-1 and Arp2/3 null is appropriate since the newly included data clearly shows the two phenotypes are not the same.

6. There is still a tremendous amount of variability in the quantification of temporal events during granule secretion (Figs 2C, 2D, 3H, 4F, and 4J). The authors draw their conclusions from the average time difference which doesn't truly represent the data in this situation.

7. The newly added circularity measurements to assay the phenomena of membrane crumpling seems out of place. Additionally, 2D measurements of the circularity of lifeact on secreting granules is highly dependent on the optical plane that is being imaged through the granule. 3D imaging on the entire secreting granule and subsequent 3D analyses is the more appropriate way to assay for curvature changes in a secreting granule.

Concerns

A. The authors provide new data demonstrating Swip-1 antibody staining in Swip-1 null glands. They conclude that "a complete loss of immunostaining in mutant glands confirmed the high specificity of the anti-Swip-1 antibody." Figure 1D shows magenta staining by the basal membrane of secretory cells. One might rather conclude that Swip-1 is no longer seen localized to secreting granules in the null.

B. The authors are still referring to the dextran experiment as a way to visualize membrane fusion (lines 225-226). As previously noted, this is incorrect. The dextran experiment identifies fusion pore formation.

<u>Reviewer 2</u>

Advance summary and potential significance to field

Hasn't changed from the first review.

Comments for the author

The revised manuscript addresses the major concerns raised in the prior review.

© 2023. Published by The Company of Biologists under the terms of the Creative Commons Attribution License (https://creativecommons.org/licenses/by/4.0/).

Second revision

Author response to reviewers' comments

Dear Michael,

Before I provide a detailed point-to-point- response, let me briefly comment on reviewer #1. Disappointingly, the reviewer mixed up the two manuscript versions, with the result that our new data are misjudged. This becomes most obvious in the first paragraph. We did not state that "*… and even after eight hours post treatment only half of the mutant glands become secretory*" and the reviewer further comments "*but figure 4B shows 100% secretion at the 8hr time point (full green bar, green secreting and black non-secreting) in the swip-1 null.*" This is an excerpt from the initial manuscript where we still used the exogenous SerpCBP-eGFP reporter, and our new data are shown in figure 3B, not in figure 4B. Overall, we have put a lot of effort into the revision and addressed all main concerns of both reviewers by establishing numerous new recombined fly stocks, in particular those expressing the SGS3-GFP reporter as a native cargo under the control of its endogenous promotor. Our new data confirmed our initial findings that loss of EFhD2/Swip-1 function impairs salivary gland secretion. Contrary to reviewer #1, these defects are not subtle but are rather very striking as shown in figure 3B. Unfortunately, the other comments by reviewer 1 are similarly less constructive than before. Below you will find our point-to-point discussion (marked in blue) including our additional data analysis.

Reviewer 1
Advance Summary and Potential Significance to Field: The summary of the advance made and potential significance to the field are identical to the primary submission

Reviewer 1
Comments for the Author: In the revised version of this manuscript Lehne and Bogdan provide several key experimental and textual changes to their primary submission. However, this revision still does not appropriately address the main concerns I have. The inconsistencies in the data and conclusions are still too numerous for publication.

Major Concerns:
1. The authors have now utilized SGS3-GFP to assay for the importance of Swip-1 to salivary gland secretion using spinning disk microscopy to visualize secretion. However, the data in figure 3B suggests that there is a subtle delay in secretion. There is 100% (within error) secretion in WT by 5 hrs and by 6 hrs in the swip-1 null. The authors state that "… and even after eight hours post treatment only half of the mutant glands become secretory" but figure 4B shows 100% secretion at the 8hr time point (full green bar, green secreting and black non-secreting) in the swip-1 null.

Unfortunately, the reviewer mixed up the initial and the revised manuscript version here. We did not state that "… and even after eight hours post treatment only half of the mutant glands become secretory" but figure 4B shows 100% secretion at the 8hr time point (full green bar, green secreting and black non-secreting) in the swip-1 null." This is an excerpt from the initial manuscript where we still used the exogenous SerpCBP-eGFP reporter. Moreover, our new data are shown in figure 3B, not in figure 4B.

We also disagree with the reviewer that defects are subtle. The defects are rather very prominent as our quantification indicates that mutant cells show only 50% (75 %) secretion after 5 hours (6 hours) compared to wild type (50 % after 2 hours; 75 % after 3 hours). Thus, these new data show a striking rather than "subtle" delayed secretion of mutant salivary glands.

Additionally, the authors state in the legends that "apoptotic and non-secreting (un- primed) SGs were discarded from analysis." If the point of the experiment is to see if glands secrete cargo, how can one discard non-secreting glands from the analysis?

© 2023. Published by The Company of Biologists under the terms of the Creative Commons Attribution License (https://creativecommons.org/licenses/by/4.0/).

Apoptotic cells should be always discarded from analysis. We indeed also excluded salivary glands that had not started secretion after 8 hours post induction and considered them unprimed to address a major concern of Reviewer #2 (see also original comments "…since the salivary glands must reach the correct developmental stage to acquire "competence" to respond to 20E, even in ex vivo culture.").

2. The authors acknowledge and include new data showing that overexpression of Swip-1 results in prolonged cargo release that is similar to the Swip-1 null phenotype "suggesting that increased EFhD2/Swip-1 protein level interferes with its function." Because this is that case the results from the Swip-1-eGFP experiments (figure 2) are compromised. Swip-1-eGFP is overexpressed to visualize when the protein is recruited to secreting cargo, related to lifeact and other markers. However, if Swip-1 overexpression prolongs the secretory event this experiment can't be compared to Zip/Lifeact, Whamy/Lifeact, Dia/Lifeact, etc.

We disagree with the reviewer. Protein recruitment/localization and function are two different things. Overexpression of Swip-1-eGFP indeed resulted in a prolonged secretion process, i.e. that the compression of the vesicle and therefore expulsion of cargo into the lumen is prolonged. However, our data do not suggest that the initial Swip-1 recruitment to the membrane is delayed but rather that it accumulates at fused secretory vesicles at early stage. Noteworthy, we also see no significant differences in recruitment between endogenously labeled Dia (GFP protein trap) and overexpressed UAS-Dia-GFP fusion (see quantification included below), which is known to interfere with endogenous protein function upon overexpression.

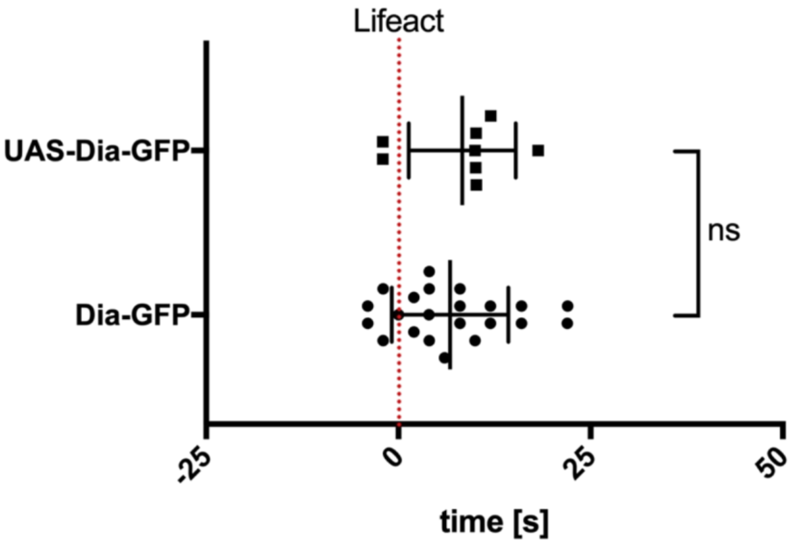

3. In figure 2C the authors conclude that Swip-1 is recruited to secreting granules before lifeact. However, in figure 2D the authors show that the time of recruitment of Swip-1 vs Arp3 is identical to that of Lifeact vs Arp3 and thus conclude both lifeact and Swip-1 are recruited simultaneously to the secreting vesicle. I am unsure how the authors come to their final conclusion here and why the data involving Arp3 supersedes that of the Swip-1/Lifeact (2C) experiment. The time relationship between Lifeact and Arp3 is a fixed linear event. If Swip- 1 is seen on granules before lifeact (2C) the same must hold true when both are individually compared to Arp3. The difference in result is likely due to variability in the analysis and large error seen in the individual replicates. See item 6 below.

From the comparison of Lifeact and Swip-1 data points we cannot conclude if there is a significant difference in time of recruitment or if the earlier mean time of Swip-1 recruitment is due to the fluctuations in our measurements, but we are within the realm of statistical possibility. We therefore had to use a third reporter (Arp3-GFP) to perform such a comparative analysis and we indeed found no significant difference as shown in figure 2D.

We disagree that "The time relationship between Lifeact and Arp3 is a fixed linear event". To our knowledge, this is not fixed but rather one observation published once in Tran et al., 2015.

© 2023. Published by The Company of Biologists under the terms of the Creative Commons Attribution License (https://creativecommons.org/licenses/by/4.0/).

4. The authors have added appropriate supplemental movies.

We thank the reviewer for pointing this out.

5. Removal of the phenotypic comparison between Swip-1 and Arp2/3 null is appropriate since the newly included data clearly shows the two phenotypes are not the same.

We agree and thank the reviewer for pointing this out.

6. There is still a tremendous amount of variability in the quantification of temporal events during granule secretion (Figs 2C, 2D, 3H, 4F, and 4J). The authors draw their conclusions from the average time difference which doesn't truly represent the data in this situation.

These are *in vivo* measurements of different endogenously and exogenously labeled- fluorescent reporters, that indeed vary in tissue as published by many different research groups. Our data does not allow the direct conclusion of the sequence of events concerning all proteins analyzed. However, we could show that Swip-1 is recruited earlier than all other examined proteins to the vesicle membrane. This is statistically significant, which we now included in figure 2C. Thus, the early recruitment of the actin-binding protein Swip-1 coincides with the polymerization of F-actin around the vesicle. In our analysis, we were not able to confirm the published data (Tran et al., 2015) even though the published method was applied and a much higher n-number of vesicles were analyzed (n of 20 to 30 SG instead of 10-12 SG, see also Tran et al., 2015).

7. The newly added circularity measurements to assay the phenomena of membrane crumpling seems out of place. Additionally, 2D measurements of the circularity of lifeact on secreting granules is highly dependent on the optical plane that is being imaged through the granule. 3D imaging on the entire secreting granule and subsequent 3D analyses is the more appropriate way to assay for curvature changes in a secreting granule.

We disagree that our new data are out of place. By contrast, we further provided new and important evidence that loss of EFhD2/Swip-1 function affects actomyosin- mediated vesicular membrane crumpling. We agree that 3D analyses could be more appropriate to characterize defective vesicular membrane crumpling. Therefore, we tried to analyze the surface to volume ratio of the vesicles during the secretion process. However, we could not obtain 3D rendering of vesicles (even with machine learning and consultation with an Imaris representative) with both enough detail of the membrane folding and complete surface structures to perform proper analysis. These problems become also very obvious in computational 4D reconstructions of secretory vesicles published by Tran et al., 2015 (see figure 1E) or Tran and Hagen 2017 (JCS, see also figure 5C). We therefore decided to perform 2D analysis to truly assess the membrane invagination opposed to comparing rather smoothened surfaces as can be seen in other publications (Tran et al., 2015, Fig. 1E; Rousso et al, 2016 Fig.1f).

Concerns

A. The authors provide new data demonstrating Swip-1 antibody staining in Swip-1 null glands. They conclude that “a complete loss of immunostaining in mutant glands confirmed the high specificity of the anti-Swip-1 antibody.” Figure 1D shows magenta staining by the basal membrane of secretory cells. One might rather conclude that Swip-1 is no longer seen localized to secreting granules in the null.

We indeed showed the high specificity of anti-Swip-1 antibody staining at the vesicular membrane (and in immunoblots as well). The remaining staining signal shown in figure 1D in magenta represents unspecific background staining of the basal surface of the salivary gland that can be often observed in many immunostainings (see also anti- pMRLC staining of the basal surface in figure 4H’. To better document this unspecific staining of the basal surface of the salivary gland, we now also included a detailed view of the basal surface in wild type and mutant salivary glands (see new figure 1C’’ and 1D’’) showing a similar unspecific signal at the basal surface in wild type and mutant SG.

© 2023. Published by The Company of Biologists under the terms of the Creative Commons Attribution License (https://creativecommons.org/licenses/by/4.0/).

B. The authors are still referring to the dextran experiment as a way to visualize membrane fusion (lines 225-226). As previously noted, this is incorrect. The dextran experiment identifies fusion pore formation.

We already changed the text accordingly. We now also deleted completely the term “membrane fusion” in the sentence “…membrane fusion (fusion pore formation) and actin recruitment …” (see also line 223).

Third decision letter

MS ID#: JOCES/2022/260366

MS TITLE: EFhD2/Swip-1 promotes exocytosis of glue granules in the exocrine Drosophila salivary gland

AUTHORS: Sven Bogdan and Franziska Lehne
ARTICLE TYPE: Research Article

I am happy to tell you that your manuscript has been accepted for publication in Journal of Cell Science, pending standard ethics checks.

© 2023. Published by The Company of Biologists under the terms of the Creative Commons Attribution License (https://creativecommons.org/licenses/by/4.0/).
